# Regional and Microenvironmental Scale Characterization of the *Zostera muelleri* Seagrass Microbiome

**DOI:** 10.3389/fmicb.2019.01011

**Published:** 2019-05-14

**Authors:** Valentina Hurtado-McCormick, Tim Kahlke, Katherina Petrou, Thomas Jeffries, Peter J. Ralph, Justin Robert Seymour

**Affiliations:** ^1^Climate Change Cluster, Faculty of Science, University of Technology Sydney, Ultimo, NSW, Australia; ^2^School of Life Sciences, Faculty of Science, University of Technology Sydney, Ultimo, NSW, Australia; ^3^School of Science and Health, Western Sydney University, Penrith, NSW, Australia

**Keywords:** seagrass microbiome, diversity, core, bacteria, microalgae, fungi, amplicon sequencing

## Abstract

Seagrasses are globally distributed marine plants that represent an extremely valuable component of coastal ecosystems. Like terrestrial plants, seagrass productivity and health are likely to be strongly governed by the structure and function of the seagrass microbiome, which will be distributed across a number of discrete microenvironments within the plant, including the phyllosphere, the endosphere and the rhizosphere, all different in physical and chemical conditions. Here we examined patterns in the composition of the microbiome of the seagrass *Zostera muelleri*, within six plant-associated microenvironments sampled across four different coastal locations in New South Wales, Australia. Amplicon sequencing approaches were used to characterize the diversity and composition of bacterial, microalgal, and fungal microbiomes and ultimately identify “core microbiome” members that were conserved across sampling microenvironments. Discrete populations of bacteria, microalgae and fungi were observed within specific seagrass microenvironments, including the leaves and roots and rhizomes, with “core” taxa found to persist within these microenvironments across geographically disparate sampling sites. Bacterial, microalgal and fungal community profiles were most strongly governed by intrinsic features of the different seagrass microenvironments, whereby microscale differences in community composition were greater than the differences observed between sampling regions. However, our results showed differing strengths of microbial preferences at the plant scale, since this microenvironmental variability was more pronounced for bacteria than it was for microalgae and fungi, suggesting more specific interactions between the bacterial consortia and the seagrass host, and potentially implying a highly specialized coupling between seagrass and bacterial metabolism and ecology. Due to their persistence within a given seagrass microenvironment, across geographically discrete sampling locations, we propose that the identified “core” microbiome members likely play key roles in seagrass physiology as well as the ecology and biogeochemistry of seagrass habitats.

## Introduction

Seagrasses are the only group of flowering plants that have fully adapted to an underwater lifestyle (Hemminga and Duarte, 2000; Larkum et al., 2018). These marine plants are an extremely valuable component of coastal ecosystems (Costanza et al., 1997; Beck et al., 2001; Orth et al., 2006), where they represent key habitat forming species (Dayton, 1972) and ecosystem engineers (Wright and Jones, 2006). Furthermore, seagrass meadows are a globally significant carbon sink, accounting for about 10% (equivalent to 27.4 Tg C year^–1^) of marine organic carbon burial (Fourqurean et al., 2012). However, the health and survival of these organisms, which are ecologically important for the value of coastal ecosystems (Costanza et al., 2014), is likely to be reliant on, or fundamentally regulated by, their association with microorganisms (Brakel et al., 2014; Brodersen et al., 2015).

It is widely recognized that plant-microbe associations are essential for the function and health of terrestrial plants (Vandenkoornhuyse et al., 2002; Berendsen et al., 2012; Hirsch and Mauchline, 2012; Schlaeppi and Bulgarelli, 2015; Vandenkoornhuyse et al., 2015), with many examples of both mutualistic and antagonistic plant–microbe interactions (Bourke, 1964; Vincent, 1980; Mylona et al., 1995; Baker et al., 1997). In the marine environment, similar close ecological associations exist between microbes and a wide range of marine benthic organisms, including corals (Rohwer et al., 2002; Bourne et al., 2009), sponges (Taylor et al., 2007; Morrow et al., 2015), and seaweeds (Egan et al., 2013; Marzinelli et al., 2015). Although less studied than terrestrial plants and other benthic eukaryotes, seagrasses also maintain intimate ecological interactions with microbial consortia living in association with the plant and within the surrounding seawater and sediments (Walters and Moriarty, 1993; Brodersen et al., 2018). For instance, microbes inhabiting seagrass leaves, roots, and rhizomes can mediate several metabolic exchanges and biogeochemical transformations that are essential for seagrass resource provision and plant growth (Hemminga et al., 1991; Hansen et al., 2000; Welsh, 2000; Cifuentes et al., 2003; Lehnen et al., 2016; Crump et al., 2018). These include sulfide oxidation (Crump et al., 2018), sulfate reduction (Hansen et al., 2000; Cifuentes et al., 2003; Lehnen et al., 2016), nitrogen fixation and nitrification (Hemminga et al., 1991; Hansen et al., 2000; Welsh, 2000; Lehnen et al., 2016), urea turnover and ammonium production (Hansen et al., 2000), sedimentation and nutrient uptake by the leaves (Harlin, 1973; Hemminga et al., 1991), and microbial consumption of plant-derived organic exudates (Harlin, 1973; Crump et al., 2018). Collectively, the microorganisms comprising the seagrass microbiome have been increasingly recognized as pivotal players in seagrass ecology (Ugarelli et al., 2017; Brodersen et al., 2018).

Spatially and temporally stable associations between a host organism and specific members of its microbial consortia are characteristic of a “core microbiome” (Astudillo-Garcia et al., 2017), comprised of a conserved assemblage of microorganisms that likely impart critical ecological functions to the host (Shade and Handelsman, 2012). The concept of the core microbiome was initially developed to understand the dynamics of bacterial communities associated with humans (Turnbaugh et al., 2009), and has since been applied to a range of host organisms and ecosystems (Hernandez-Agreda et al., 2017). The composition of a host organism’s core microbiome can be governed by both the intrinsic physiology of the host and external environmental factors (Marzinelli et al., 2015). In benthic marine organisms like sponges, core microbiomes can be both highly species-specific (Schmitt et al., 2012) and conserved across large biogeographical scales (Schmitt et al., 2012; Thomas et al., 2016). In many host organisms, discrete core microbiomes occur in association with different organs, tissues or other morphological features of the host (Huttenhower et al., 2012; Keenan et al., 2013). For example, in corals, discrete core microbiomes are associated with the coral branches, the surface mucus layer, intracellular spaces within tissues and the skeletal matrix (Rohwer et al., 2002; Hernandez-Agreda et al., 2016). Similarly, in terrestrial plants discrete core microbiomes are associated with different plant features, including the phyllosphere (i.e., above-ground aerial surfaces of plants), endosphere (i.e., root interior), and rhizosphere (i.e., zone around the root that is influenced by the plant) (Lindow and Brandl, 2003; Knief et al., 2012; Lundberg et al., 2012; Coleman-Derr et al., 2016).

Microbial assemblages associated with seagrasses inhabit a number of discrete microenvironments within the plant, including the phyllosphere, the endosphere and the rhizosphere (Ugarelli et al., 2017). Levels of photosynthesis, oxygen and the diffusive exchange of organic substrates vary across the seagrass phyllosphere, from the upper leaf to the leaf sheath (Larkum et al., 2007; Hogarth, 2015; Rubio et al., 2017), creating marked small-scale spatial heterogeneity in microenvironmental conditions for leaf associated microorganisms. Below the sediment surface, the roots and rhizomes anchor the plant into the sediment and mediate nutrient uptake, while also mediating chemical exchanges with microorganisms through the exudation of dissolved organic material into the rhizosphere (Hemminga and Duarte, 2000; Badri and Vivanco, 2009; Hogarth, 2015; Koren et al., 2015; Kuzhiumparambil et al., 2017). Levels of oxygen and organic substrates within the rhizosphere are generally highly dissimilar to the surrounding sediments (Koren et al., 2015), promoting microscale heterogeneity in microbial abundance, activity and community composition (Brodersen et al., 2018). Hence, while often closely located, the different physical and chemical conditions within discrete seagrass microenvironments are likely to favor the growth of disparate microbial assemblages and underpin small-scale partitioning in the composition and function of seagrass-associated microbial communities.

Seagrass microbiomes have previously been shown to differ above and below the sediment surface (Crump and Koch, 2008; Mejia et al., 2016; Ettinger et al., 2017), as well as between the seagrass and the adjacent seawater and sediment (Jensen et al., 2007; Gordon-Bradley et al., 2014; Cucio et al., 2016; Fahimipour et al., 2017; Martin et al., 2018). In addition to this small-scale heterogeneity, seagrass microbiomes have also been shown to vary across larger, regional scales, whereby microbiological properties are driven by local environmental conditions (Uku et al., 2007; Jiang et al., 2015; Bengtsson et al., 2017; Ettinger et al., 2017; Fahimipour et al., 2017; Crump et al., 2018). For instance, the microbial assemblages associated with *Zostera marina*, *Zostera noltii*, and *Cymodocea nodosa* have been shown to vary over continental scales (Cucio et al., 2016; Fahimipour et al., 2017). Observations to date indicate that the seagrass microbiome is a product of both localized intrinsic features of specific plant microenvironments and larger scale environmental drivers. However, a unified understanding of the factors determining the structure of the seagrass microbiome and the spatial and temporal scales over which these communities are governed by specific features of the seagrass environment is lacking.

Here, we aim to elucidate the significance of microenvironmental and regional forces in shaping the microbiome of the seagrass species *Zostera muelleri* (*Z. muelleri*). We compared bacterial, microalgal, and fungal communities associated with six different plant microenvironments, including the upper and lower leaf, the sheath, the roots and rhizomes, surficial sediment, and adjacent seawater across four spatially discrete habitats, with the goal of understanding the nature and dynamics of the *Z. muelleri* microbiome.

## Materials and Methods

### Field Survey

**FIGURE 1:**
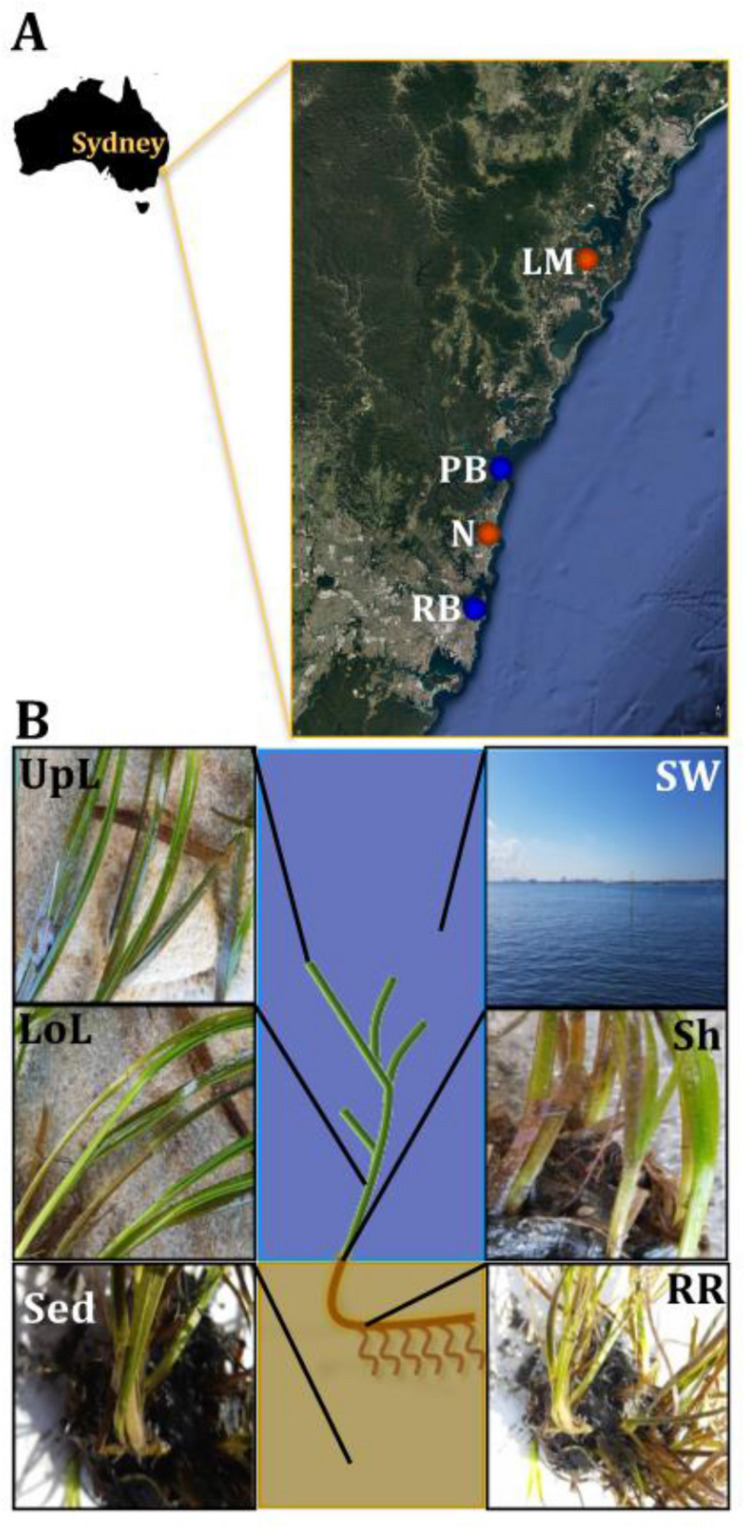
Study sites and sampling strategy. Samples were collected between October and November 2015 across a region spanning 86 km of coastline in NSW, Australia **(A)**. Study sites included coastal (blue) and estuarine (orange) habitats, which were selected in accordance to habitat feature data (e.g., proximity to contamination sources and human activities) that was subsequently coupled with environmental parameters (e.g., water temperature and salinity) and genetic markers of anthropogenic pollution (i.e., *intI1*) to rank sites according to their specific conditions and level of impact. For details of the study sites selection criteria and categorization, see Supplementary Figure 1 and Supplementary Table 1. At each site, samples from six different microenvironments within the plant (black fonts) and its surroundings (white fonts) were collected, based on the variety of conditions offered by these different niches **(B)**. UpL, upper leaf; LoL, lower leaf; Sh, sheath; RR, roots and rhizomes; Sed, sediment; SW, seawater; PB, Palm Beach; RB, Rose Bay; N, Narrabeen Lagoon; LM, Lake Macquarie.

Samples associated with the seagrass species *Z. muelleri* were collected from two coastal and two estuarine habitats, across a region spanning 86 km of coastline in New South Wales (NSW), Australia (Figure 1A). These included, Palm Beach (33°35′15.8″S 151°19′25.0″E), Rose Bay (33°52′20.1″S 151°15′43.7″E), Lake Macquarie (33°09′29.4″S 151°31′54.9″E) and Narrabeen Lagoon (33°43′11.0″S 151°17′40.4″E). Our four sampling locations were chosen as distinct, yet representative habitats colonized by seagrass meadows in NSW (Green and Short, 2003), a region characterized by significant seagrass cover in both coastal and estuarine environments. Narrabeen Lagoon is a semi-enclosed lagoon and Lake Macquarie is an estuary, and both differed from our two open coastal habitats (i.e., Palm Beach and Rose Bay) with respect to depth distribution, salinity, and seawater nutrient concentrations. Other differences among all sites include different extent of water inflows from the open-ocean, terrestrial runoff, and levels of anthropogenic impact due to human activities.

Sample collection took place between October and November 2015, with all sites surveyed during low-tide conditions (<2 m depth). Water physicochemical properties (i.e., temperature and conductivity as indicative of salinity) were measured *in situ* using a multi-probe meter (WTW Multi 3430, Germany). At each site, samples were collected from six microenvironments associated with different features of the plant (Figure 1B). These included: (i) the upper and (ii) lower parts of the leaf, (iii) the sheath, (iv) roots and rhizomes, (v) surrounding sediment, and (vi) seawater. We considered these six microenvironments best represented seagrass morphology and anatomy, despite the wide phenotypic plasticity found between populations and species.

### Sampling Protocols

A highly standardized sampling protocol was used to collect samples from the seagrass (i.e., leaves and roots and rhizomes) and the surrounding microenvironments (i.e., surficial sediment and adjacent seawater). Individual specimens of *Z. muelleri* (i.e., total biomass) were collected with sterile-gloved hands from at least two physically separated meadows (i.e., well-defined area of a dense group of plants) per site, to account for potential differences between meadows. Sampled plants were homogeneously distributed across the meadows chosen and collected with a minimum distance of 20 cm between plants. Each shoot was pulled out from the substrate, ensuring all plant sections were intact and then placed onto a clean tray to separate surficial sediment (i.e., sediment adjacent to the roots and rhizomes). For each plant, 1 g of sediment was taken adjacent to the roots and rhizomes from 1 to 3 cm under the surface using a syringe, subsequently homogenized in a clean tray to ensure the detachment of plant material and other contaminants, and immediately placed into 1.8 mL Nunc^®^ CryoTubes. Once sediment was collected, each plant was rinsed with seawater collected on site, and placed into a Ziploc^®^ plastic bag filled to 2/3 of its total volume with the same water. In addition, 10 L of seawater was collected from the surface waters of the sampling site using Nalgene bottles; all replicate seawater samples were obtained from within ∼30 cm of the seagrass. After collection, seagrass (*n* = 5), sediment (*n* = 5), and seawater samples (*n* = 3) were transported to the laboratory on ice and immediately processed upon arrival.

In the laboratory, each plant was gently rinsed free of adhering sediment with Milli-Q water (Millipore Corporation, Billerica, MS, United States) to avoid excess accumulated debris on the periphyton layer (i.e., mixture of microbes and detritus attached to submerged surfaces). Plant material was successively divided into four microenvironments (upper leaf, lower leaf, sheath, and roots and rhizomes) with sterile scissors and scalpels. For each tissue type, 5 biological replicates were collected, comprising a surface area of 2.5 cm^2^ for leaves, 0.5 cm^2^ for sheaths, and a volume of 2 mL for entire branched roots and rhizomes, in order to keep enough distance between leaf fractions and to collect the required 0.25 g of sample for DNA extractions. Once processed and placed into 2 mL Nunc^®^ CryoTubes, seagrass and sediment samples were immediately snap frozen in liquid nitrogen and stored at −80°C prior to analysis. Water samples were kept on ice until triplicate 2 L samples were immediately filtered onto 0.2 μm polycarbonate membrane filters (Millipore) using a peristaltic pump upon return to the laboratory. Filters were snap frozen and stored at −80°C.

### DNA Extraction

For the leaf, sheath, roots and rhizomes, and sediment samples, genomic DNA was extracted from 0.25 g of plant tissue or sediment, using a bead beating and chemical lysis-based DNA extraction kit (PowerSoil^®^ DNA Isolation Kit, MoBio Laboratories, Carlsbad, CA, United States). Microbial DNA from water samples was extracted from filters using the PowerWater^®^ DNA isolation Kit (MoBio Laboratories, Carlsbad, CA, United States). Both kits were used in accordance with the manufacturer’s standard protocol. DNA quantity and purity were evaluated using a Nanodrop-1000 spectrophotometer (Thermo Fisher Scientific, NanoDrop Products, Wilmington, DE, United States).

### Bacterial Community Characterization

To examine bacterial community composition within all samples, the 16S rRNA gene was amplified with the universal forward primer 27F (5′-AGAGTTTGATCMTGGCTCAG-3′) and the universal reverse primer 519R (5′-CGGTTACCTTGTTACGACTT-3′) (Weisburg et al., 1991). PCR reactions were performed in 25 μL volumes containing 12.5 μL GoTaq Green Master Mix, 0.4 μL of each primer (10 μM), and 2 μL of template DNA. PCR cycling conditions involved an initial activation step at 95°C for 120 s, followed by 30 cycles of: denaturation at 95°C for 30 s, annealing at 50°C for 30 s and extension at 72°C for 90 s, followed by a holding stage at 72°C for 10 min. The resultant amplicons were visualized on 1% agarose gel with GelRed (1:10000). Genomic DNA was used to prepare DNA libraries with the Illumina TruSeq DNA library preparation protocol. Sequencing was performed on the Illumina MiSeq platform (at Molecular Research LP, Shallowater, TX, United States) following the manufacturer’s guidelines. Subsequently generated raw data files were deposited in the Sequence Read Archive (SRA) under BioProject number PRJNA342246 (Hurtado-McCormick, 2018a).

### Fungal Community Characterization

In order to characterize fungal community composition, we used Illumina Miseq profiling of internal transcriber spacer (ITS) markers. Specifically, the ITS2 region was amplified by targeting a site in the 5.8S encoding gene with the fITS7 (5′-GTGARTCATCGAATCTTTG-3′)/ITS4 (5′-TCCTCCGCTTATTGATATGC-3′) primer set (Ihrmark et al., 2012). PCR reactions were performed as follows: initial activation step of 94°C for 5 min, followed by 35 cycles of: denaturation at 94°C for 30 s, annealing at 50°C for 30 s, and extension at 72°C for 30 s, followed by a holding stage at 72°C for 7 min. Sequencing was performed on the Illumina MiSeq platform (at the Next Generation Genome Sequencing Facility of Western Sydney University). Raw data files in FASTQ format were deposited in the Sequence Read Archive (SRA) under BioProject number PRJNA493529 (Hurtado-McCormick, 2018b).

### Sequence Data Analysis

Bacterial 16S rRNA gene sequences were analyzed using a customized pipeline (Kahlke, 2018). Briefly, paired-end DNA sequences were de-multiplexed using MOTHUR, v1.39.0 (Schloss et al., 2009), then joined using FLASH, v1.2.11 (Magoc and Salzberg, 2011), quality-filtered using MOTHUR, and finally de-replicated using VSEARCH, v2.3.2 (Rognes et al., 2016). Quality filtering involved both, trimming of ambiguous bases in each of the sequences, as well as removal of short fragments with low quality scores from the data set. Operational Taxonomic Units (OTUs) were defined at 97% sequence identity and subsequently clustered using VSEARCH. The same tool was also used to detect and remove chimera sequences based on curated sequences from the Greengenes database, released on 13/08/2013 (DeSantis et al., 2006), and to build the OTU table. Taxonomy assignments were performed using BLAST, vBLAST+ (Altschul et al., 1990), in QIIME, v1.9.1 (Caporaso et al., 2010) to generate a representative set of OTUs that was aligned against the Greengenes database. Sequences were rarefied to the same depth (2380 sequences per sample) to remove the effect of sampling effort upon analysis (Supplementary Table 9). Given the nature of this study’s experimental design and the importance of replication in complex data-sets, the rarefaction cut-off was chosen to include at least triplicates per sample type.

Microalgal communities were identified from a secondary taxonomic assignment performed on sequences classified as “chloroplast” by the Greengenes classification obtained from the 16S rRNA analysis of bacterial communities (Needham and Fuhrman, 2016). A separate OTU table was generated by BLASTn search of the PhytoREF database, downloaded July 01, 2015 (Decelle et al., 2015), which was used to provide a phylogenetic characterization of chloroplast sequences. This OTU table was subsequently screened to exclude sequences classified as plants or macroalgae, and finally relative abundances of microalgae were re-calculated for each OTU from previously rarefied data.

Initial sequence processing for fungal ITS genes was conducted using QIIME, v1.9.1 (Caporaso et al., 2010). Briefly, low-quality regions were trimmed from the 5′ end of sequences, and paired ends were joined with fastq-join (Aronesty, 2011, 2013) and de-multiplexed. Sequences containing ambiguous bases were removed from the dataset along with low-quality reads and chimeric sequences. Referenced-based chimera detection (Nilsson et al., 2015) was performed using the UCHIME algorithm from the USEARCH package (Edgar, 2010; Edgar et al., 2011) implemented within VSEARCH, v2.3.2 (Rognes et al., 2016). OTUs were defined as clusters of 97% sequence similarity using UCLUST (Edgar, 2010). The resultant OTU table was filtered to remove singletons and seagrass-affiliated sequences. OTU sequences were screened for non-fungal sequences using BLAST (Altschul et al., 1990), against the nucleotide database from the National Center for Biotechnology Information (NCBI). Non-fungal sequences were identified using BASTA (Kahlke and Ralph, 2019) and the following parameters: -l 250 (sequence length), -m 0 (mismatches), and -i 97 (identity). These sequences were subsequently removed from the dataset. Final taxonomies were assigned to the filtered OTU set (i.e., sequences of unknown origin) using the UNITE database v6.9.7 (Koljalg et al., 2013), BLAST, and vBLASTC (Altschul et al., 1990). Finally, the resultant filtered OTU table was rarefied to an even number of sequences per samples to ensure equal sampling depth (i.e., lower number of sequences per sample = 1456). Given the nature of this study’s experimental design and the importance of replication in complex datasets, the rarefaction cut-off was chosen to include at least triplicates per sample type (Supplementary Table 9). Due to the low number of fungal sequencing reads from the leaf that remained after removal of putative seagrass sequences, only the mycobiomes associated with seagrass roots and rhizomes, sediments and seawater were used for further post-sequencing analyses, while the seagrass leaf samples were omitted from cross-sample comparisons. A separate re-analysis of these samples with unrarefied data supported all of the scientific conclusions of our original manuscript, except for the predominance of the Rhytismataceae family in the upper leaf mycobiome, which instead represented rare taxa (relative abundance < 1% in all samples) within seagrass-associated fungal communities.

### Post-sequencing Analyses

Alpha diversity was estimated by calculating the Chao1 and Shannon’s diversity indices in QIIME, v1.9.1 (Caporaso et al., 2010). The exponential function was applied to the Shannon’s diversity index to calculate the true Shannon’s diversity (i.e., effective number of species) in accordance to the approaches used by Lundberg et al. (2012) to estimate alpha diversity of bacterial communities associated with the rhizosphere (including surrounding sediments) and the endophytic compartment of the model, terrestrial plant *Arabidopsis thaliana* (*A. thaliana*). Permutational Multivariate Analysis of Variance (PERMANOVA) was used to test the statistical significance of the differences between and within microenvironments and sites, separately, in a nested design. These statistical analyses were performed in PRIMER-E, v7 (Clarke, 1993; Clarke et al., 2014; Clarke and Gorley, 2015).

Differences in community composition (i.e., beta diversity) were characterized using non-parametric multi-dimensional scaling (nMDS). PERMANOVA was used to test for statistical significance of the differences between and within microenvironments and sites. In order to further characterize the significant differences observed between sites within each microenvironment, we performed hierarchical CLUSTER analyses (Timm, 2002). Each of these analyses were performed in PRIMER-E, v7 (Clarke, 1993; Clarke et al., 2014; Clarke and Gorley, 2015).

To identify “discriminatory OTUs” between microenvironments, we coupled pair-wise analyses of Similarity Percentages (SIMPER) (Clarke, 1993) performed in PRIMER-E, v7 (Clarke, 1993; Clarke et al., 2014; Clarke and Gorley, 2015), with extensive hypothesis testing of taxonomic profiles using Kruskal–Wallis-H and Tukey–Kramer statistical tests performed in Statistical Analysis of Metagenomic Profiles (STAMP, v2.1.3) (Parks et al., 2014). Significantly over-represented OTUs with the highest contributions to the differences between microenvironments were defined as “discriminatory OTUs,” with exceptions including non-significantly over-represented OTUs with consistent high contributions.

A custom script was used for the selection of core microbiomes (Kahlke, 2017). Core microbiomes were defined for each microenvironment and for the entire leaf (i.e., pooling the three phyllosphere microenvironments) in accordance with the approaches used by Lundberg et al. (2012) to define the core microbiome of the endophitic compartment within the bacterial communities in the rhizosphere of *A. thaliana*. In order to account for possible outliers in the data, any OTU present (relative abundance > 0%) in two out of three biological replicates within a given site (occurrence ≥ 67%), across all four sites, was classified as a core OTU. Abundant (greater than 1%) pelagic microbes were removed from the phyllosphere core microbiomes to eliminate the influence of possible sampling artifacts.

### Study Site Characterization Using *IntI1*

To characterize the putative level of anthropogenic influence experienced by seagrasses in each of the four study environments, quantitative PCR (qPCR) was used to quantify the relative abundance of the clinical class 1 integron-integrase gene (*intI1*), which has previously been demonstrated to be a good proxy for anthropogenic pollution (Gillings et al., 2015). Serial dilutions of a plasmid harboring the *intI1* gene amplified from an environmental sample (seawater collected at Botany Bay, NSW, Australia) were used as a template to generate a standard curve. All samples and the standard curve were run in the same plate, which was prepared by an epMotion^®^ 5075l Automated Liquid Handling System and conducted on a BIO-RAD CFX384 Touch^TM^ Real-Time PCR Detection System^TM^ (Bio-Rad Laboratories, Inc., Hercules, CA, United States). We used the BIO-RAD CFX Manager software to estimate *intI1* gene copies for triplicate reactions per sample (*n* = 12). Each 5 μL reaction consisted of 2.5 μL of iTaq UniverSYBR Green SMX 2500^®^ (Bio-Rad Laboratories, Inc., Hercules, CA, United States), 0.1 μL of nuclease free water, 0.2 μM of the forward primer int1.F (5′-GGGTCAAGGATCTGGATTTCG-3′), 0.2 μM of the reverse primer int1.R (5′-ACATGCGTGTAAATCATCGTCG-3′) (Mazel et al., 2000) and 2 μL of diluted (1:5) DNA template. The qPCR was subsequently run under the following thermal cycling conditions: initial denaturation for 3 min at 95°C, followed by 39 cycles of denaturation for 15 s at 95°C and annealing and extension for 1 min at 60°C. Coupling the results of this analysis with the measured environmental parameters and habitat feature data, allowed us to categorize our sampling locations into four levels of anthropogenic impact.

## Results and Discussion

### Characterization of Sampling Sites

We coupled measurements of physicochemical parameters (Supplementary Table 1) and quantification of a genetic marker for anthropogenic pollution (*intI1*, Supplementary Figure 1) (Gillings et al., 2015) to categorize our four study sites based on an anthropogenic impact ranking (Supplementary Table 1), highlighting the disparate conditions of the sampled seagrass habitats. Based on our categorization, Narrabeen Lagoon was the most impacted site (i.e., highest level of influence from human activities), followed by Rose Bay and Lake Macquarie, whereas Palm Beach was the most pristine site. However, given the highly dynamic nature of coastal/estuarine environments, where conditions can change markedly on short time periods, our sampling events represent discrete snap-shots in time that lack historical information about the prior conditions of the environment, and therefore we suggest caution regarding the use of this information to infer the drivers of microbiome structure.

### The Seagrass Bacterial Microbiome

We investigated bacterial community composition and diversity in six discrete seagrass microenvironments associated with *Z. muelleri* across the four different sampling locations (Figure 1), in order to: (i) characterize the seagrass microbiome, (ii) determine the variability and/or level of conservation of the seagrass microbiome across different spatial scales (i.e., plant microenvironments and the region), and (iii) identify persistent, or “core” microorganisms within the seagrass microbiome. Using 16S rRNA gene sequencing, we contrasted patterns in alpha- and beta- diversity of bacterial assemblages among seagrass microenvironments and sites differing in physicochemical properties of the seawater (i.e., temperature and salinity), exposure to the open ocean (coastal vs. estuarine habitats) and anthropogenic impact (Supplementary Figure 1 and Supplementary Table 1).

**FIGURE 2:**
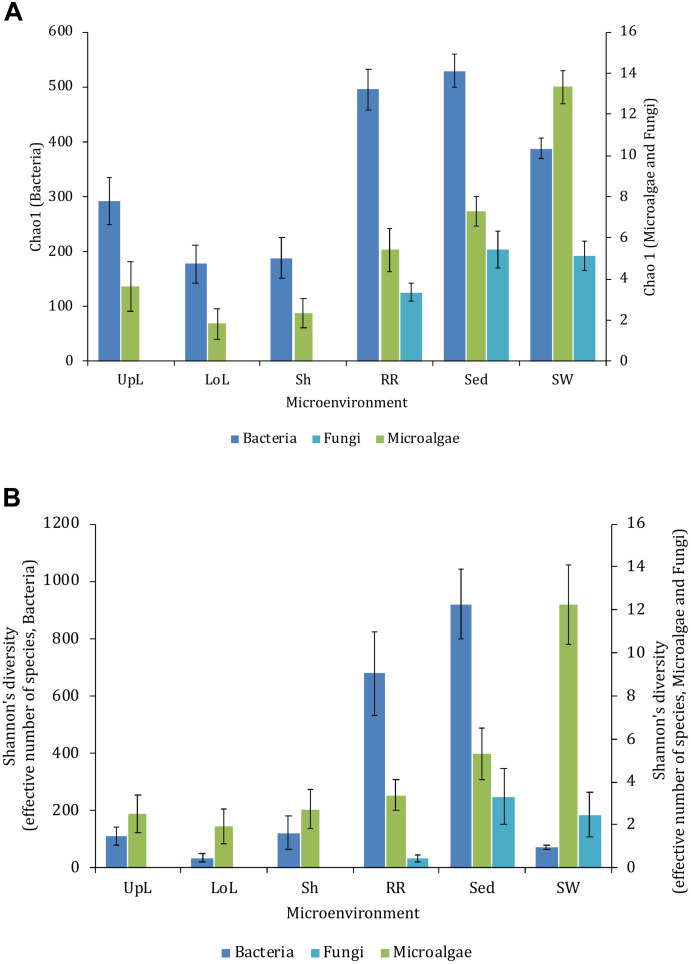
Microbial mean alpha diversity across seagrass microenvironments. Multiple comparisons between Chao1 diversity **(A)** and Shannon’s diversity index **(B)**, calculated for each taxa and microenvironment separately, were tested for statistical significance with Permutational Multivariate Analysis of Variance (PERMANOVA, Minkowski metric distance matrix and nested design). Mean values for each microenvironment are shown, and error bars reflect the standard error of the mean.

Alpha diversity, as measured by Chao1 and Shannon’s diversity index, varied significantly both between sampling locations (p_Chao1_ = 0.0017, p_Shannon__’__s_ = 0.0001) and seagrass microenvironments (*p* = 0.0001). However, *post hoc* analyses for both diversity indices (Supplementary Table 2) indicated that the between site differences were solely driven by differences between Rose Bay and all other sites (*p* < 0.05), with the exception of Lake Macquarie (p_Chao1_ = 0.1495). Alpha diversity levels within the three microenvironments within the phyllosphere (i.e., upper leaf, lower leaf, and the sheath) did not differ statistically from one another or between sites (*p* > 0.05), with the exception of the upper leaf and the sheath at Narrabeen Lagoon (p_Chao1_ = 0.0465). The bacterial assemblages inhabiting the roots and rhizomes and the sediments were the most diverse microenvironments (Figure 2), which might be a consequence of higher levels of microscale heterogeneity and persistence of biogeochemical gradients within this zone (Stapel and Hemminga, 1997; Evrard et al., 2005; Jensen et al., 2007; Brodersen et al., 2018; Fraser et al., 2018).

**FIGURE 3:**
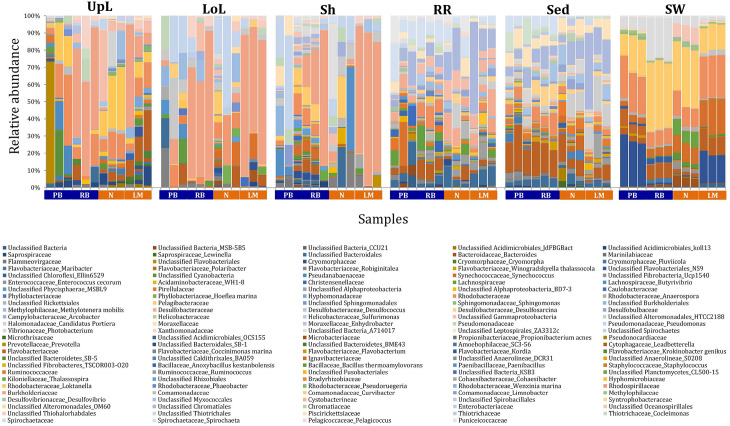
Bacterial community composition across seagrass microenvironments. Beta diversity of bacterial microbiomes across the six microenvironments within the plant and its surroundings. Triplicate samples per microenvironment within each of the four sampling sites (*n* = 72) are colored by the highest assigned taxonomic level. Unique OTUs were summarized at the species level, and the representation of taxonomic groups within each sample are plotted. Only representative species with a relative abundance >1% in all samples are shown to help remove visual clutter. UpL, upper leaf; LoL, lower leaf; Sh, sheath; RR, roots and rhizomes; Sed, sediment; SW, seawater; PB, Palm Beach; RB, Rose Bay; N, Narrabeen Lagoon; LM, Lake Macquarie.

**FIGURE 4:**
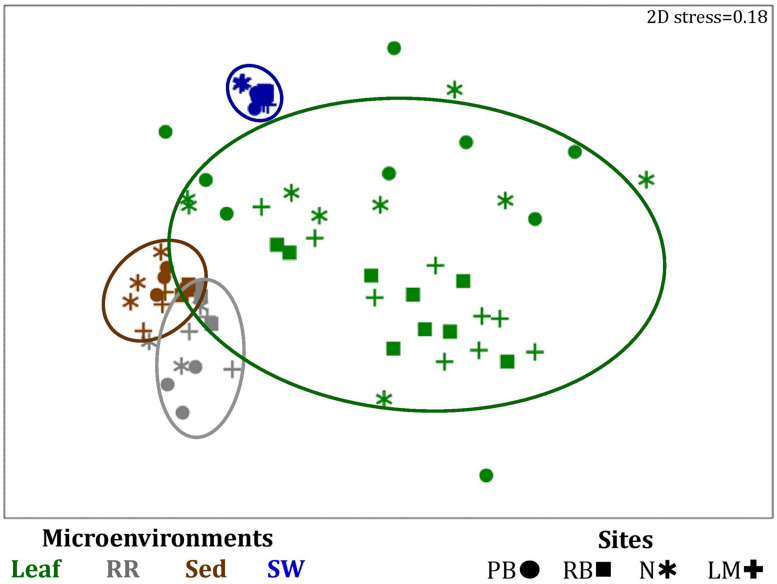
Microenvironmental and regional partitioning of the seagrass bacterial microbiome. Non-parametric multidimensional scaling (nMDS) of bacterial microbiomes (*n* = 72), based on a lower triangular resemblance calculated with the S17 Bray-Curtis similarity measure from relative abundances of OTUs (high values down-weighted with square root). Samples are colored by microenvironment (Leaf: upper and lower sections, RR, roots and rhizomes; Sed, sediment; SW, seawater), with different shapes for sites (PB, Palm Beach; RB, Rose Bay; N, Narrabeen Lagoon; LM, Lake Macquarie). Sample clustering patterns by microenvironment are shown in ellipses in the nMDS plot, representing the level of similarity between samples based on the degree to which OTUs are shared between them. The 2D stress is shown in the upper right corner of the nMDS plot (Kruskal stress formula = 1, minimum stress = 0.01). The nMDS for the three microenvironments within the phyllosphere is provided in Supplementary Figure 3 and a hierarchical cluster analysis (CLUSTER) for all samples is provided in Supplementary Figure 2.

Significant variability (*p* = 0.0001) in bacterial assemblage structure occurred between seagrass microenvironments (Figure 3), which was apparent in both multi-dimensional scaling plots (nMDS, Figure 4) and dendograms (Supplementary Figure 2), whereby clear clustering of bacterial community composition between specific seagrass microenvironments was evident within each sampling site, except for the three microenvironments within the leaf. Similar to the patterns in alpha diversity, significant differences in bacterial assemblage structure were also observed between study sites (*p* = 0.0001), supporting the influence of local environmental forces on the seagrass microbiome. However, the differences in bacterial assemblage structure between microenvironments were greater than those between the sampling regions. Despite the spatial separation of just a few centimeters across an individual plant, the microbial communities from the different microenvironments showed the greatest variability, with only 42% shared bacterial taxa, whereas at the regional scale, where plants were separated by up to 52 km (i.e., largest distance between sites) and subject to differing local environmental conditions, sampling locations shared a higher proportion of 58% of bacterial taxa (ECV, Supplementary Table 3).

**FIGURE 5:**
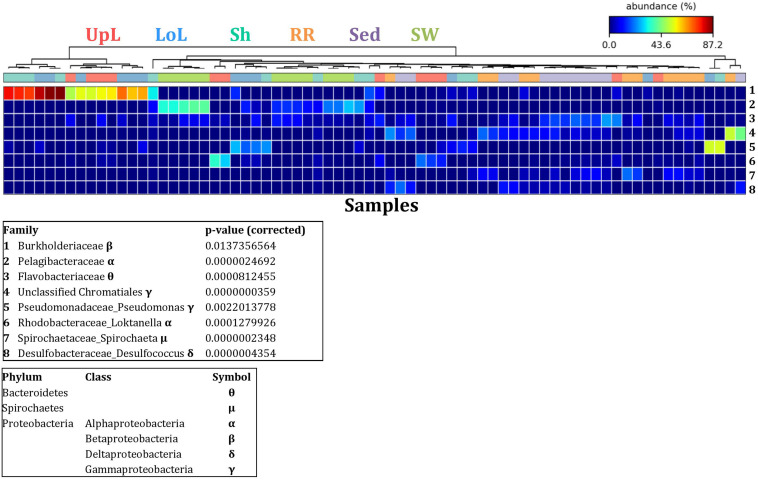
Bacterial discriminatory OTUs at the microenvironmental scale. Extensive hypothesis testing of taxonomic profiles was coupled with similarity percentages analyses (SIMPER) for bacterial microbiomes across the six microenvironments. The proportion of sequences (mean frequency %) of OTUs significantly over-represented (Kruskal–Wallis *H*-test, α = 0.05, effect sizes: η^2^) and consistently contributing to the differences between microenvironments is indicated by varying color intensities. Corrected *p*-values were calculated using the Benjamini–Hochberg’s approach. Two-way crossed SIMPER analyses were performed with site and microenvironment variables as factors (S17 Bray–Curtis similarity matrix). High contributors were selected from the top-5 contributors of each pair-wise comparison between microenvironments, and those OTUs consistently accounting for the dissimilarities between any given microenvironment and at least three other microenvironments were chosen as high contributors to couple with the statistical results. High contributors that were significantly over-represented were classified as discriminatory OTUs (i.e., 1–8). OTUs are sorted by decreasing mean abundance, and samples are clustered by average neighbor distance (UPGMA, distance threshold = 0.75) and colored by microenvironment. Different symbols represent the distribution of enriched phyla. UpL, upper leaf; LoL, lower leaf; Sh, sheath; RR, roots and rhizomes; Sed, sediment; SW, seawater.

To further explore the key drivers of the variability within bacterial structures across different seagrass microenvironments and geographical locations, we coupled similarity percentages community analysis (SIMPER) (Clarke, 1993; Clarke et al., 2014; Clarke and Gorley, 2015) with extensive hypothesis testing of taxonomic profiles using Kruskal-Wallis-H and Tukey-Kramer statistical tests (Parks et al., 2014). Using this combined approach, we found 8 discriminatory OTUs that were (i) significantly over-represented in a given microenvironment (*p* < 0.03), and/or (ii) among the top five contributors to the observed dissimilarities between microenvironments as determined by SIMPER. These OTUs spanned three bacterial phyla, including four classes of the Proteobacteria (Figure 5).

The clear clustering of seawater samples on nMDS (Figure 4) was principally driven by the dominant bacteria within these samples, corresponding to the families Pelagibacteraceae (12 unique OTUs), Rhodobacteraceae (139 unique OTUs), and Cryomorphaceae (20 unique OTUs), which made up 26, 17, and 11% of these communities, respectively (Figure 3). These families, along with a member of the Halomonadaceae (*Candidatus* Portiera sp., 7 unique OTUs), discriminated seawater samples from the other microenvironments (Figure 5). This is consistent with *Pelagibacter*, and other members of the SAR11 clade, being the dominant bacteria in seawater communities (Morris et al., 2002; Bowman et al., 2012; Brown et al., 2012; Giovannoni, 2017), and members of the Rhodobacteraceae, Halomonadaceae, and Cryomorphaceae often dominating pelagic microbial assemblages in coastal and estuarine habitats (Pinhassi et al., 2004; Prabagaran et al., 2006; Buchan et al., 2014; Jeffries et al., 2016).

The bacterial assemblages inhabiting the seagrass sediments also represented a clearly distinguished cluster from the other microenvironments on the nMDS, with conservation of the bacterial assemblage structure within this microenvironment across all four sampling sites (Figure 4 and Supplementary Figure 2). Within this microenvironment, OTUs matching the Flavobacteriaceae (116 unique OTUs), the order Chromatiales (39 unique OTUs), and the Desulfobacteraceae (*Desulfococcus* sp., 26 unique OTUs) dominated these communities, accounting for 13, 13, and 8% of the sequences, respectively (Figure 3). The relative over-representation of these organisms within the sediment was also most responsible for the differences in bacterial assemblage structure relative to the other five microenvironments (Figure 5). Members of these three taxa have previously been shown to dominate the sediments associated with seagrasses (Sun et al., 2015; Cucio et al., 2016, 2018; Ettinger et al., 2017) and salt marsh plants (Thomas et al., 2014), where Chromatiales and Desulfobacteraceae play important roles in nutrient cycling, given their sulfur-oxidizing and sulfate-reducing capabilities, respectively (Kleindienst et al., 2014; Varon-Lopez et al., 2014). Members of the Flavobacteriaceae are also often abundant in coastal marine sediments when sufficient oxygen is available (Raulf et al., 2015; Sun et al., 2015), where they can play a prominent role in the degradation of complex polymeric substrates (i.e., organic matter decomposition) (Bowman et al., 2012).

Like the communities associated with the surrounding microenvironments, bacterial assemblages within the roots and rhizome samples collected across the four sites generated a discrete cluster, discriminated from the other microenvironments on nMDS (Figure 4). The bacterial community in the roots and rhizomes was significantly different from the microbiomes associated with the surrounding sediments and seawater across all four sampling locations (*p* < 0.05, Supplementary Table 3). There were also statistically significant differences between these samples and each of the three phyllosphere microenvironments at all sites (*p* < 0.05 for 10 comparisons), with only two exceptions at Palm Beach (p_sh_ = 0.0776) and Narrabeen Lagoon (p_lol_ = 0.0510) (Supplementary Table 3). Relative to the other five microenvironments, the roots and rhizomes were characterized by a higher proportion of unclassified members of the orders Chromatiales (39 unique OTUs) and Bacteroidales (63 unique OTUs) and the Spirochaetaceae (*Spirochaeta* sp., 41 unique OTUs) (Figure 3). The same OTUs from the Chromatiales that dominated sediment communities were also over-represented in the roots and rhizomes relative to the phyllosphere and surrounding seawater, and along with the spirochaetes, these bacteria drove the differences between this community and the other five microenvironments (Figure 5). Although not often found directly in association with the roots and rhizomes, members of the Spirochaetaceae are often found within seagrass sediments (Cifuentes et al., 2000; Doty, 2015; Trevathan-Tackett et al., 2017), while members of the Bacteroidales have elsewhere been shown to dominate communities attached to roots of aquatic angiosperms (Crump and Koch, 2008).

In contrast to the clear discrimination of bacterial assemblages in the other microenvironments, the three microenvironments within the phyllosphere (i.e., upper leaf, lower leaf, and sheath) overlapped with one another on the nMDS plot (Supplementary Figure 3), but were clearly discriminated from the bacterial assemblages from the roots and rhizomes and the surrounding seawater and sediments (Figure 4). Furthermore, there were no statistical differences in bacterial community structure between these three compartments of the phyllosphere at Palm Beach and Rose Bay (*p* > 0.05), whereas only the upper leaf and the sheath differed from each other at Narrabeen Lagoon (*p* = 0.0411) and Lake Macquarie (*p* = 0.0282) (Supplementary Table 3). It should be noted, however, that the lack of statistical differences between the three microenvironments within the phyllosphere could have resulted from either a more homogenous distribution of bacteria across the entire phyllosphere or from the high level of heterogeneity observed across replicates within each site (Figure 3). Therefore, while no statistically significant differences were observed between the three different compartments of the phyllosphere, there remains the possibility that a higher degree of replication may have resolved significant differences, given the spatially variable photosynthetic rates and nutrient contents throughout the leaf (Duarte, 1990; Hemminga et al., 1991; Stapel and Hemminga, 1997; Borum et al., 2006; Larkum et al., 2007; Koren et al., 2015), and the dissimilar oxic conditions between the sheath and the upper leaf (Tyerman et al., 1984).

Across all sampling locations, a single family, the Burkholderiaceae (2 unique OTUs), dominated all three microenvironments within the phyllosphere, representing an average of 30% of these communities (Figure 3). Some OTUs, however, were exclusively dominant in a single phyllosphere microenvironment. These included OTUs matching the Rhodobacteraceae in the upper leaf (including *Loktanella* sp., 139 unique OTUs, average relative abundance = 13%), the Comamonadaceae in the lower leaf (9 unique OTUs, relative abundance = 6%), and the Paenibacillaceae in the sheath (*Paenibacillus* sp., 1 unique OTU, relative abundance = 6%). The assemblage structure of phyllosphere-associated bacteria differed from the other microenvironments primarily due to an over-representation of the Burkholderiaceae (2 unique OTUs) in both the lower leaf and the sheath, the Rhodobacteraceae (*Loktanella* sp., 14 unique OTUs) in the upper leaf, and the Pseudomonadaceae (*Pseudomonas* sp., 4 unique OTUs) in the lower leaf. Together, these bacteria drove the differences between the phyllosphere to the rhizosphere and the adjacent seawater (Figure 5).

Overall, these results show that while the nature of the seagrass microbiome is influenced to some extent by local environmental conditions that can vary with biogeography, intrinsic differences between the discrete microenvironments associated with the host have a larger effect on shaping the seagrass microbiome structure (Figures 3, 4 and Supplementary Figure 2). Some regional differences in the overall bacterial assemblage structure between sampling locations (Supplementary Table 3) were potentially governed by environmental characteristics at each site, such as physicochemical conditions, exposure to the open ocean and anthropogenic impact (Supplementary Figure 1 and Supplementary Table 1). However, the observed conservation of some bacterial OTUs within specific microenvironments across all sites, is highly suggestive of the existence of universally important members of the microbiome across all environments (Shade and Handelsman, 2012; Astudillo-Garcia et al., 2017). To explore this pattern more directly, we next determined the existence of core microbiome members within the specific seagrass microenvironments.

### The Bacterial Core Microbiomes

**FIGURE 6:**
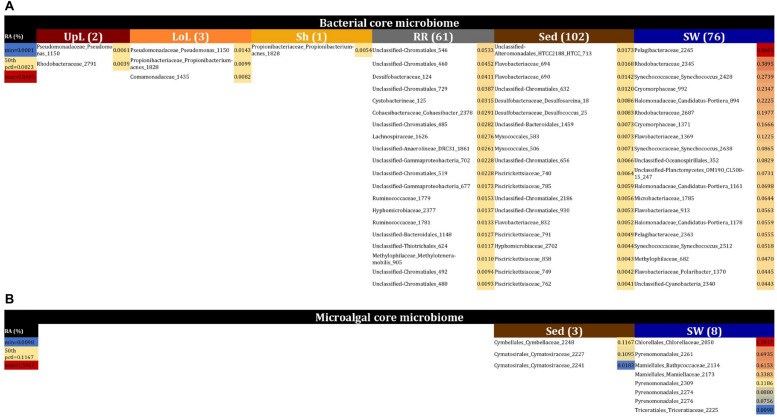
The seagrass core microbiomes. Bacterial **(A)** and microalgal **(B)** core OTUs associated with the seagrass and surrounding microenvironments were identified based on their predominance (i.e., occurrence ≥ 67%, relative abundance > 0%) across the four sampling sites. Cores are listed under columns for each microenvironment, and their sizes are shown in brackets. Core OTUs were identified at the family (bacteria) and order (microalgae) levels and are colored by their relative abundance within each microenvironment. Abundant pelagic microbes were removed from the phyllosphere core microbiomes, and only the 20 most abundant bacterial core OTUs within each microenvironment are plotted to help remove visual clutter. The full version of the table is provided in Supplementary Table 4. UpL, upper leaf; LoL, lower leaf; Sh, sheath; RR, roots and rhizomes; Sed, sediment; SW, seawater; RA, relative abundance.

No single OTU was observed across all seagrass microenvironments, which both indicates that there is not an overall “core seagrass bacterial microbiome” and confirms that the seagrass microenvironments examined here represent markedly different microbial niches. However, core microbiome members were found in each of the microenvironments, whereby there was evidence of maintenance of specific core members across the four discrete sampling regions. The size of core microbiomes varied substantially between microenvironments, ranging from one core OTU within the sheath microenvironment, up to 102 core OTUs within the surrounding sediments (Figure 6A). The core microbiome members of the six seagrass microenvironments cumulatively spanned more than 39 bacterial families, across 14 phyla (Supplementary Table 4).

The upper leaf microenvironment was characterized by a core community including two OTUs from the Alpha- and Gamma- Proteobacteria, which together made up 75% of the core within this microenvironment. This is consistent with previous observations, whereby members of these groups represented ≥50% of bacterial communities associated with the seagrass phyllosphere (Weidner et al., 2000; Jiang et al., 2015), and both classes are widely recognized as abundant bacteria within the *Zostera* microbiomes (Cucio et al., 2016; Bengtsson et al., 2017; Ettinger et al., 2017; Fahimipour et al., 2017; Crump et al., 2018). More specifically, these two core OTUs were classified as the Pseudomonadaceae and Rhodobacteraceae families and made up 46 and 29% of the upper leaf core, respectively (Figure 6A and Supplementary Table 4). Notably, the Pseudomonadaceae include pathogens and leaf epiphytes of terrestrial angiosperms (Hirano and Upper, 2000) and have also been shown to dominate the microbiomes of seagrass leaves from geographically linked coastal locations (Jiang et al., 2015). The Rhodobacteraceae are common pelagic and surface-associated marine bacteria that incorporate a broad suite of metabolisms, including chemoorganotrophy and photoheterotrophy (Dixon and Kahn, 2004; Haselkorn and Kapatral, 2005), with some members known to produce antibacterial compounds that may influence leaf surface colonization by other microbes, including pathogens (Dang et al., 2008). Members of this family are commonly observed on the leaves of seaweeds (Fernandes et al., 2012) and seagrasses (Crump and Koch, 2008; Mejia et al., 2016; Ettinger et al., 2017), and particularly in *Z. marina* (Crump and Koch, 2008; Ettinger et al., 2017). Their relative abundance previously demonstrated to be linked to specific features of the host (i.e., different compartments and health status) or environmental conditions (i.e., water turbidity, nutrients, and geomorphological features). Members of this group have also been implicated in macrophyte pathogenesis due to their increased abundance in aged and bleached macroalgal phenotypes (Fernandes et al., 2012; Zozaya-Valdes et al., 2015; Mancuso et al., 2016).

The core bacterial assemblage inhabiting the lower leaf exhibited similarities to that of the upper leaf and sheath microenvironments, with an OTU from the Pseudomonadaceae overlapping with the upper leaf core and another OTU from the Propionibacteriaceae coinciding with the only core member of the sheath microbiome (Figure 6A and Supplementary Table 4). The classical propionibacteria have been traditionally isolated from dairy products, but there are also strains isolated from soils and terrestrial plants (Stackebrandt et al., 2006), and even from different areas of the human body (McGinley et al., 1978). These microorganisms are known as a ubiquitous family within coral- (Kuang et al., 2015) and cone snail-associated microbiomes (Valliappan et al., 2014). While not previously reported in seagrasses, other bacteria within the higher taxonomic rank, the Actinobacteria, have been repeatedly observed dominating the communities associated with the seagrass leaf and the rhizosphere (Cucio et al., 2016; Mejia et al., 2016; Bengtsson et al., 2017; Fahimipour et al., 2017; Crump et al., 2018; Ugarelli et al., 2019). When firstly defining the three phyllosphere cores, we observed that shared members across these three groups (i.e., phyllosphere microenvironments) included OTUs from the Pelagibacteraceae and Synechococcaceae families. Given that *Pelagibacter* and *Synechococcus* are both ubiquitous and dominant members of pelagic microbial assemblages (Li, 1998; Giovannoni and Stingl, 2005), it is probable that their consistent occurrence on leaf surfaces represented a sampling artifact. This was also supported by our observations of OTUs from the Pelagibacteraceae and Synechococcaceae dominating the core associated with the surrounding seawater, and therefore we removed abundant pelagic microbes from the phyllosphere datasets in order to analyze the phyllosphere core microbiomes.

The core microbiome of the roots and rhizomes included 61 bacterial OTUs (Figure 6A and Supplementary Table 4). Among these were a large number of core OTUs from the Chromatiales (18% of total core OTUs in the roots and rhizomes), Desulfobacteraceae (7% of total core OTUs in the roots and rhizomes), and Rhodobacteraceae (7% of total core OTUs in the roots and rhizomes). Members of the Desulfobacteraceae family are anaerobic, chemolithotrophic microorganisms, commonly involved in sulfate reduction and nitrogen fixation processes in seagrass environments, particularly near to the roots and rhizomes (Welsh et al., 1996; Welsh, 2000; Bagwell et al., 2002; Lovell, 2002; Devereux, 2013; Sun et al., 2015; Cucio et al., 2016; Lehnen et al., 2016; Ettinger et al., 2017; Crump et al., 2018). Moreover, these bacteria are well-known abundant microorganisms within *Zostera* microbiomes, where they discriminate communities associated with roots from those associated with the leaf and surrounding sediments (Ettinger et al., 2017). Therefore, we suggest that core members from the Desulfobacteraceae are nitrogen fixers within the rhizosphere of *Z. muelleri*. The Chromatiales are members of a large group of purple sulfur bacteria (Overmann, 1997; Storelli et al., 2013) that are commonly observed in sediments surrounding *Zostera* meadows and salt marshes (Thomas et al., 2014). Co-habitation of sulfate reducing and sulfur oxidizing bacteria within seagrass rhizomes has been observed elsewhere (Cifuentes et al., 2000; Crump and Koch, 2008; Cucio et al., 2016, 2018), whereby sulfur oxidizing bacteria are likely to play an essential role in the detoxification of sulfides produced by the sulfate reducing bacteria (Cifuentes et al., 2000; Crump and Koch, 2008; Cucio et al., 2016, 2018; Fahimipour et al., 2017).

Together, these results provide evidence of a clear differentiation of core bacterial communities across the different microenvironments within the seagrass, instead of a unified seagrass core microbiome. The phyllosphere core microbiome mainly consisted of Alpha- and Gamma- Proteobacterial OTUs exploiting the oxic conditions and high levels of labile organic substrates within the leaf surface microenvironment, whereas the core microbiome of the roots and rhizomes included, likely sulfate reducing, members of the Deltaproteobacteria. The persistence of core microbiomes across the seagrass microenvironments has not previously been explored at this level of detail. However, our demonstration of discrete core microbiomes across the different seagrass microenvironments is consistent with patterns in terrestrial plants, whereby the rhizosphere, the phyllosphere and the root and leaf endospheres host communities that are both distinct from one other and the surrounding soils (Coleman-Derr et al., 2016). The patterns observed here are also consistent with other benthic marine organisms including corals, where distinct microbial communities colonize different microhabitats within the coral colony, coral polyps and coral tissue (Ainsworth et al., 2009).

### The Seagrass Microalgal Microbiome

Microalgal communities within seagrass meadows collectively provide crucial ecosystem services, including the contribution of considerable levels of primary production and energy transfer to higher trophic levels (Stafford-Bell, 2016). Consistent with the patterns observed for bacteria, levels of alpha diversity among microalgal communities varied significantly between both seagrass microenvironments (*p* = 0.0001) and sites (p_Chao1_ = 0.0327, p_Shannon__’__s_ = 0.0001) (Supplementary Table 5). However, *post hoc* analyses for both diversity indices indicated that the between site differences were solely driven by differences between Palm Beach and Lake Macquarie (p_Chao1_ = 0.0018, p_Shannon__’__s_ = 0.0001). Although no consistent patterns were observed across habitats, several significant differences in alpha diversity were observed between seagrass microenvironments within each site (Supplementary Table 5). In general, microalgal diversity within the seagrass microenvironments was lower (*p* < 0.05) than in the surrounding seawater (Figure 2), which might be attributed to competitive interactions between microalgae and other epiphytes on the seagrass leaves, and/or regulatory mechanisms whereby microalgae are suppressed by metabolic products from the host (Harlin, 1975; Pinckney and Micheli, 1998).

**FIGURE 7:**
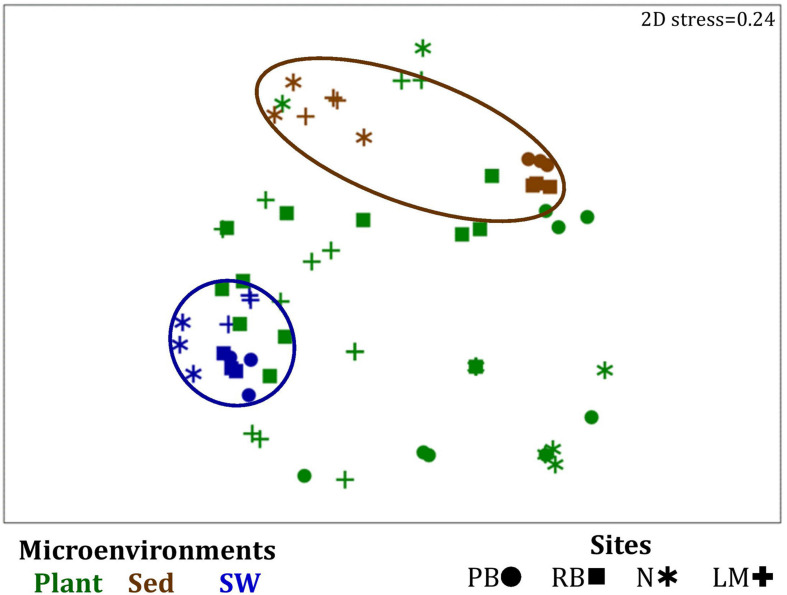
Microenvironmental and regional partitioning of the seagrass microalgal microbiome. Non-parametric multidimensional scaling (nMDS) of microalgal microbiomes (*n* = 72), based on a lower triangular resemblance calculated with the S17 Bray–Curtis similarity measure from relative abundances of OTUs (high values down-weighted with square root). Samples are colored by microenvironment (Plant: upper leaf, lower leaf, sheath and roots and rhizomes; Sed, sediment, SW, seawater), with different shapes for sites (PB, Palm Beach; RB, Rose Bay; N, Narrabeen Lagoon; LM, Lake Macquarie). Sample clustering patterns by microenvironment are shown in ellipses in the nMDS plot, representing the level of similarity between samples based on the degree to which OTUs are shared between them. The 2D stress is shown in the upper right corner of the nMDS plot (Kruskal stress formula = 1, minimum stress = 0.01). A hierarchical cluster analysis (CLUSTER) for all samples is provided in Supplementary Figure 4 and the nMDS for the four microenvironments associated with the plant is provided in Supplementary Figure 5.

Similarly to the bacteria, the composition of microalgal assemblages varied significantly between both seagrass microenvironments (*p* = 0.0001) and sampling sites (*p* = 0.0001). Although statistically significant differences in microalgal composition were observed between sampling sites (*p* < 0.05), the differences between microenvironments within each sampling location were greater [ECV, Mi(Si) = 488.81, Supplementary Table 6]. However, the clear partitioning within the assemblage structure that was observed for bacteria across the different seagrass microenvironments on nMDS plots was not as evident for microalgae, with only the surrounding seawater and sediment associated communities generating clearly discrete clusters (Figure 7 and Supplementary Figure 4). Furthermore, unlike the seagrass-associated bacterial communities, the nature of the variability in microalgal structure across microenvironments differed between habitats. Explicitly, there were no significant differences between microalgal communities associated with different plant microenvironments (i.e., phyllosphere and roots and rhizomes, Supplementary Figure 5) at Rose bay and Lake Macquarie (*p* > 0.05), whereas the roots and rhizomes assemblages differed significantly from the lower leaf communities at Palm Beach and Narrabeen Lagoon (*p* < 0.05) and also from the upper leaf at Narrabeen Lagoon (*p* = 0.0482) (Supplementary Table 6).

**FIGURE 8:**
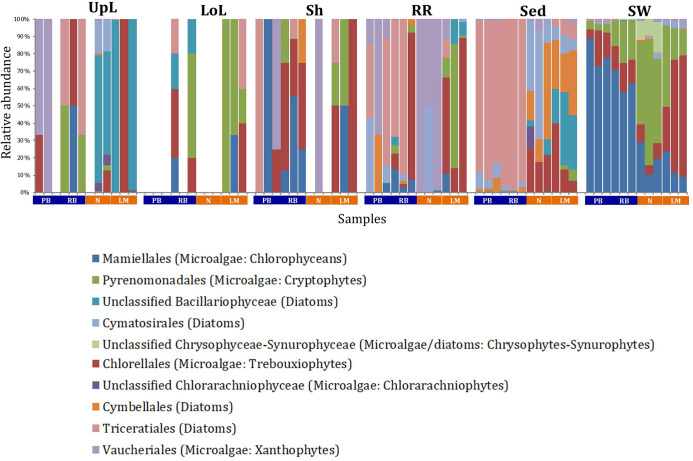
Microalgal community composition across seagrass microenvironments. Beta diversity of microalgal microbiomes across the six microenvironments within the plant and its surroundings. Triplicate samples per microenvironment within each of the four sampling sites (*n* = 72) are colored by taxonomic order. Unique OTUs were summarized at the species level, and the representation of taxonomic groups within each sample are plotted. Only representative species with a relative abundance >1% in all samples are shown to help remove visual clutter. UpL, upper leaf; LoL, lower leaf; Sh, sheath; RR, roots and rhizomes; Sed, sediment; SW, seawater; PB, Palm Beach; RB, Rose Bay; N, Narrabeen Lagoon; LM, Lake Macquarie.

A single and ubiquitous order of green microalgae, namely the Chlorellales (3 unique OTUs), dominated the microalgal community across all microenvironments and sites, representing on average 23% of these assemblages, with the exception of the surrounding sediments (Figure 8). The sediments, on the other hand, were exclusively dominated by the Cymatosirales (2 unique OTUs) and the Cymbellales (1 unique OTU), comprising 14% and 16% of the sequences within all sediment samples, respectively.

**FIGURE 9:**
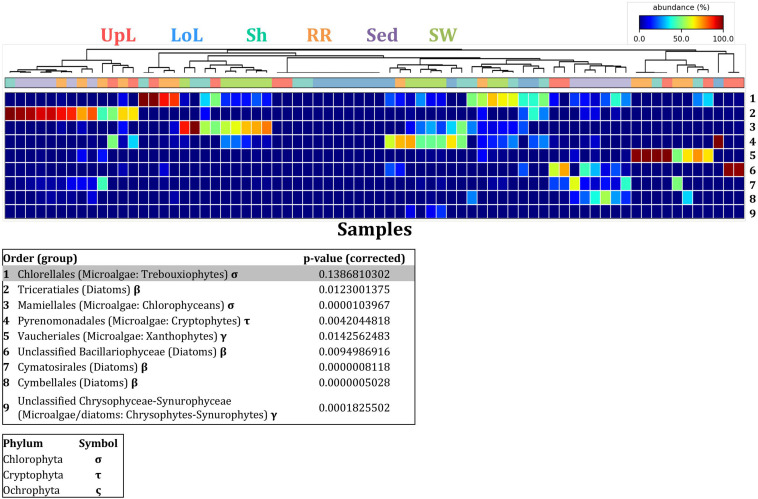
Microalgal discriminatory OTUs at the microenvironmental scale. Extensive hypothesis testing of taxonomic profiles was coupled with similarity percentages analyses (SIMPER) for microalgal microbiomes across the six microenvironments. The proportion of sequences (mean frequency %) of OTUs significantly over-represented (Kruskal–Wallis *H*-test, α = 0.05, effect sizes: η^2^) and consistently contributing to the differences between microenvironments is indicated by varying color intensities. Corrected p-values were calculated using the Benjamini–Hochberg’s approach. Two-way crossed SIMPER analyses were performed with site and microenvironment variables as factors (S17 Bray–Curtis similarity matrix). High contributors were selected from the top-5 contributors of each pair-wise comparison between microenvironments, and those OTUs consistently accounting for the dissimilarities between any given microenvironment and at least three other microenvironments were chosen as high contributors to couple with the statistical results. High contributors that were significantly over-represented were classified as discriminatory OTUs (i.e., 1–9). OTUs are sorted by decreasing mean abundance, and samples are clustered by average neighbor distance (UPGMA, distance threshold = 0.75) and colored by microenvironment. Different symbols represent the distribution of enriched phyla. High contributors that were not significantly over-represented (gray) were also classified as discriminatory OTUs if their contribution to the differences between microenvironments was consistent. UpL, upper leaf; LoL, lower leaf; Sh, sheath; RR, roots and rhizomes; Sed, sediment; SW, seawater.

In the upper leaf microenvironment, members of the Bacillariophyceae (4 unique OTUs) were the dominant microalgae, comprising groups known to contain both benthic and pelagic representatives (Crosby and Wood,1958a,b). This family represented an average of 33% of sequences and was responsible for the greatest differentiation from the other microenvironments, where it made up <9% of the microalgal assemblage (Figure 9). The Bacillariophyceae include diatoms, commonly among the most abundant and productive phototrophic microbes associated with seagrasses (Jacobs and Noten, 1980; Govindasamy and Anantharaj, 2013; Ambo-Rappe, 2016). Compositional changes of epiphytic diatoms, including members of the Bacillariophyceae, are closely related to morphological changes of the seagrass leaf (Chung and Lee, 2008). These differences in species composition and the specific modifications of the blade surface itself might alter competitive interactions between major algal groups (Pinckney and Micheli, 1998).

The roots and rhizomes were dominated by OTUs affiliated with the Vaucheriales (2 unique OTUs) and Triceratiales (1 unique OTU) orders, which represented 29% and 24% of the sequences across all locations, respectively (Figure 8). The Triceratiales include benthic and epontic diatom species, with representatives previously isolated from corals, fossil beds, marine mud, seagrasses, and similar aquatic plants (Crosby and Wood, 1958b). Notably, this order, along with several members of the Bacillariophyceae, have been shown to be major components of the epiphytic diatom community in other seagrass species (López-Fuerte et al., 2013). The Vaucheriales are yellow-green algae that have also been widely observed as epiphytes in salt marshes, seagrass meadows and mangroves (Gallagher and Humm, 1981; Saifullah et al., 2003).

Our results provide evidence for microenvironmental partitioning of the seagrass microalgal microbiome, with often clear differences in the identity of microalgal OTUs dominating different microenvironments. However, and in contrast to our observations for bacterial assemblages, no core microalgal members of the seagrass microbiome were observed for any of the plant-associated microenvironments, indicating a lower level of geographic conservation of these patterns. Core microalgal microbiomes were only identified for the sediment and seawater microenvironments (Figure 6B). The sediment-associated microbiome included three core members belonging to the orders Cymbellales (1 OTU) and Cymatosirales (2 OTUs), whereas the seawater-associated microbiome comprised eight core members matching the orders Chlorellales (1 OTU), Pyrenomonadales (4 OTUs), Mamiellales (2 OTUs), and Triceratiales (1 OTU). The absence of any clear “core microalgal microbiome” within *Z. muelleri* perhaps implies a weaker ecological coupling between seagrasses and specific microalgal taxa, relative to that observed for the bacterial component of the seagrass microbiome.

### The Seagrass Mycobiome

Although less studied in seagrasses, several fungi have been demonstrated to be highly beneficial for aquatic and terrestrial plant fitness while establishing intimate relationships with their host (i.e., mycorrhizal associations) to facilitate nutrient uptake or compete against other potentially pathogenic microbes (Azcon-Aguilar et al., 1999; Kohout et al., 2012; Raghukumar, 2012). In this study, fungal communities associated with *Z. muelleri* displayed significantly different levels of alpha diversity for the two measured indices (Chao1 and Shannon’s Index) between both seagrass microenvironments (*p*_Chao1_ = 0.0006, *p*_Shannon’s_ = 0.0001) and sampling sites (*p*_Chao1_ = 0.0051, *p*_Shannon’s_ = 0.0010) (Supplementary Table 7). Our post-hoc analyses indicated that the differences across habitats were mostly driven by differences between Lake Macquarie and all other sites. At Lake Macquarie, fungal microbiomes exhibited significantly lower levels of alpha diversity (*p* < 0.05, Supplementary Table 7). Moreover, several significant differences in alpha diversity were observed between seagrass microenvironments within each site (Supplementary Table 7). Even though some of these differences varied from site to site, general patterns were similar to those observed for microalgal assemblages, whereby seagrass-associated microenvironments (here roots and rhizomes only) had lower levels of fungal diversity than the surrounding seawater and sediments (Figure 2). This is possibly due to antifungal chemical defenses and physiological responses from the host against co-occurring marine fungi, which have been well described for other seagrass species (Ross et al., 2008).

**FIGURE 10:**
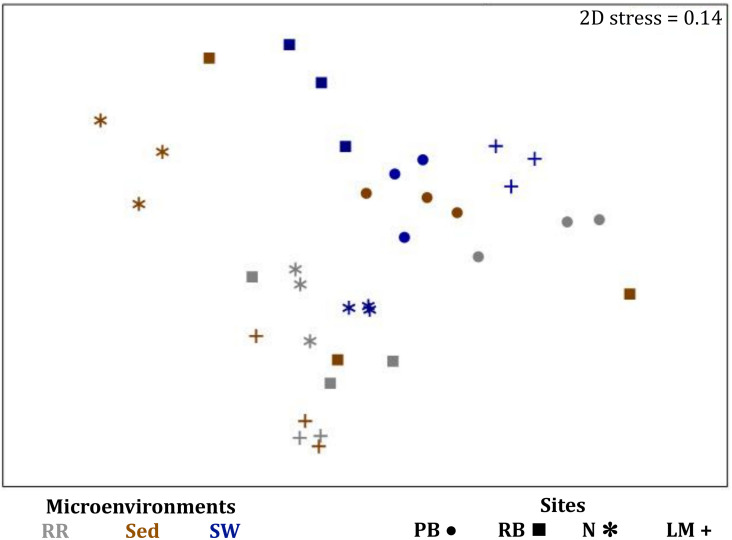
Microenvironmental and regional partitioning of the seagrass fungal microbiome. Non-parametric multidimensional scaling (nMDS) of fungal microbiomes (*n* = 35), based on a lower triangular resemblance calculated with the S17 Bray–Curtis similarity measure from relative abundances of OTUs (high values down-weighted with square root). Samples are colored by microenvironment (RR, roots and rhizomes; Sed, sediment, SW, seawater), with different shapes for sites (PB, Palm Beach; RB, Rose Bay; N, Narrabeen Lagoon; LM, Lake Macquarie). Sample clustering patterns by microenvironment within each site represent the level of similarity between samples based on the degree to which OTUs are shared between them. The 2D stress is shown in the upper right corner of the nMDS plot (Kruskal stress formula = 1, minimum stress = 0.01). The nMDS for the three microenvironments associated with the leaf is provided in Supplementary Figure 6 and a hierarchical cluster analysis (CLUSTER) is provided in Supplementary Figure 7.

Consistent with the patterns observed for bacterial and microalgal assemblages, fungal community structure varied significantly across both seagrass microenvironments (*p* = 0.0001) and sampling sites (*p* = 0.0001). Notably, all sites differed significantly from each other (*p* < 0.05). However, the differences between microenvironments within each sampling location explained a greater level of variation between mycobiomes compared to the differences between sites [ECV, Mi(Si) = 1262.70, Supplementary Table 8]. Roots and rhizomes, sediment and seawater communities formed discrete, separated clusters within each site, as evidenced in nMDS (Figure 10) and CLUSTER (Supplementary Figure 7) analyses. However, such clear separation of fungal communities between microenvironments was not apparent in unrarefied data from leaf samples (Supplementary Figure 6).

**FIGURE 11:**
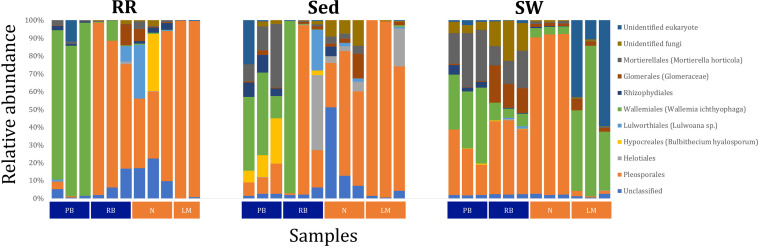
Fungal community composition across seagrass microenvironments. Beta diversity of fungal microbiomes across the three seagrass microenvironments studied here. Triplicate samples per microenvironment within each of the four sampling sites (*n* = 35) are colored by the highest assigned taxonomic level. Unique OTUs were summarized at the species level, and the representation of taxonomic groups within each sample are plotted. Only representative species with a relative abundance > 1% in all samples are shown to help remove visual clutter. RR, roots and rhizomes; Sed, sediment; SW, seawater; PB, Palm Beach; RB, Rose Bay; N, Narrabeen Lagoon; LM, Lake Macquarie.

**FIGURE 12:**
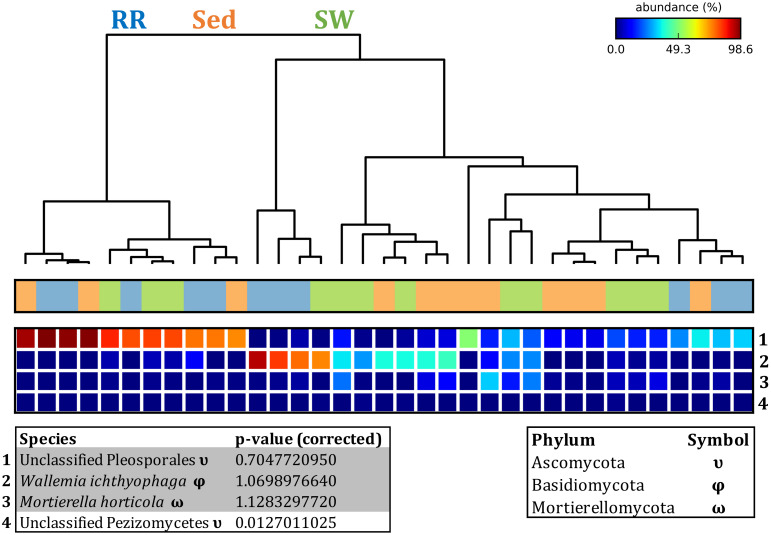
Fungal discriminatory OTUs at the microenvironmental scale. Extensive hypothesis testing of taxonomic profiles was coupled with similarity percentages analyses (SIMPER) for fungal microbiomes across microenvironments. The proportion of sequences (mean frequency %) of OTUs significantly over-represented (Kruskal–Wallis *H*-test, α = 0.05, effect sizes: η^2^) and consistently contributing to the differences between microenvironments is indicated by varying color intensities. Corrected *p*-values were calculated using the Benjamini–Hochberg’s approach. Two-way crossed SIMPER analyses were performed with site and microenvironment variables as factors (S17 Bray–Curtis similarity matrix). High contributors were selected from the top-5 contributors of each pair-wise comparison between microenvironments, and those OTUs consistently accounting for the dissimilarities between any given microenvironment and the other two microenvironments were chosen as high contributors to couple with the statistical results. High contributors that were significantly over-represented were classified as discriminatory OTUs (i.e., 1–4). OTUs are sorted by decreasing mean abundance, and samples are clustered by average neighbor distance (UPGMA, distance threshold = 0.75) and colored by microenvironment. Different symbols represent the distribution of enriched phyla. High contributors that were not significantly over-represented (gray) were also classified as discriminatory OTUs if their contribution to the differences between microenvironments was consistent. RR, roots and rhizomes; Sed, sediment; SW, seawater.

Fungal OTUs identified within four taxonomic groups consistently dominated fungal assemblages across the three microenvironments and four sampling locations studied here (Figures 11, 12). This is consistent with the hypothesis of extreme ecological flexibility acclaimed for obligate marine fungal species (Nicoletti and Andolfi, 2018). OTUs matching the order Pleosporales (291 unique OTUs) and the species *Wallemia ichthyophaga* (54 unique OTUs) represented the most abundant fungi across the roots and rhizomes, sediments and seawater microenvironments, making up an average of 38 and 18% of these communities, respectively (Figures 11, 12). Many freshwater and marine species of Pleosporales have been described to date, including several endophytes and saprophytes of plants, as well as symbionts, parasites and pathogens of seagrasses and marine macroalgae (Suetrong et al., 2009; Zhang et al., 2009; Boonmee et al., 2012; Hyde et al., 2013; Hashimoto et al., 2017). Some species are also dominant members of microbiomes associated with mangroves, showing a microenvironmental preference for intertidal parts of the host, which occur above the water level (Raghukumar, 2012). Our observations of predominant Pleosporales OTUs across all three microenvironments and particularly within roots and rhizomes, where these fungi represented 55% of the mycobiome, are highly consistent with previous reports of the dominance of a single marine fungus from the Pleosporales, probably representing a new genus, associated with the roots of the seagrass species *Posidonia oceanica* (Vohník et al., 2016). While, to our knowledge, the other dominant fungal species, *W. ichthyophaga*, has not previously been reported in seagrasses, it has been found to occur in association with other benthicmarine organisms, including corals (Raghukumar, 2012). We also observed OTUs that dominated the microenvironments surrounding the seagrass. These included the species *Mortierella horticola* (42 unique OTUs) and unclassified members of the Pezizomycetes class (7 unique OTUs), which represented 7 and 0.38% of the sediment and seawater fungal communities, respectively (Figures 11, 12). Despite its low relative abundance, the Pezizomycetes was the only taxon that differed significantly across the three seagrass microenvironments (*p* = 0.013, Figure 12). Nevertheless, further exploration of our beta diversity data revealed high relative abundances and high contributions to microenvironmental dissimilarities of the other fungi aforementioned, suggesting their potential importance within the seagrass mycobiome.

Besides the Pleosporales and *W. ichthyophaga*, we observed additional OTUs that were consistently present in all plant microenvironments, with the exception of the upper leaf. These included members of the Glomeraceae family (133 unique OTUs), which were consistently found in the lower leaf, sheath, and roots and rhizomes, where they represented an average of 9% of these assemblages (Figures 11, 12). The Glomeraceae are arbuscular mycorrhizal fungi, known for their obligate, symbiotic association with the roots of vascular plants (Schubler et al., 2001). While the lack of mycorrhizal symbioses in seagrasses has been previously proposed (Nielsen et al., 1999), our observations of the consistent presence of the Glomeraceae within the mycobiomes associated with lower, achlorophyllous parts of the seagrass, across all sampling locations, suggest a potentially important role of this fungus in the *Z. muelleri* mycobiome.

Operational Taxonomic Units belonging to the Rhytismataceae family (53 unique OTUs) were consistently present only in the upper leaf and accounted for 4% of these communities (Figures 11, 12). While we only observed Rhytismataceae in the leaf, and not in the roots and rhizomes or the surrounding sediments, members of this group have been previously isolated from the rhizosphere in other seagrass species (Panno et al., 2013; Gnavi et al., 2014). As many endophytes of the foliar communities in wood plants (Ganley et al., 2004), they may represent substantial, unknown biodiversity with functional novelties.

Here we chose to use 97% similarity criteria for defining fungal OTUs characterized using our ITS sequencing approach, which we consider a suitable conservative approach given the lower levels of taxonomic diversity covered in fungal ITS databases (relative to e.g., bacteria) and is consistent with values previously used to characterize the mycobiome associated with terrestrial plants (Giordano et al., 2009) and coastal grasses (Sánchez-Márquez et al., 2008). The overall dominance of four taxonomic groups across the three microenvironments studied here is in line with previous observations of very narrow mycobiomes associated with seagrasses (Devarajan and Suryanarayanan, 2002; Vohník et al., 2016), plants from salt marshes (Al-Nasrawi and Hughes, 2012), mangroves (Xing and Guo, 2011), and other aquatic plants (Kohout et al., 2012). Nevertheless, and similar to microalgae, we did not observe a conserved “core” of fungal associates within any of the seagrass microenvironments (here roots and rhizomes only), and a core fungal microbiome of two members was only identified for the seawater microenvironment. These core OTUs belonged to the family Glomeraceae and the order Pleosporales. Our results are indicative of a weaker ecological coupling between seagrasses and fungal taxa, relative to that observed for seagrass bacterial interactions. We propose that, relative to bacteria, which appear to display highly specific interactions with different components of the plant due to a stronger influence of the conditions at the microscale, seagrass-associated fungi appear to establish more generalist relationships with their host.

## Conclusion

**FIGURE 13:**
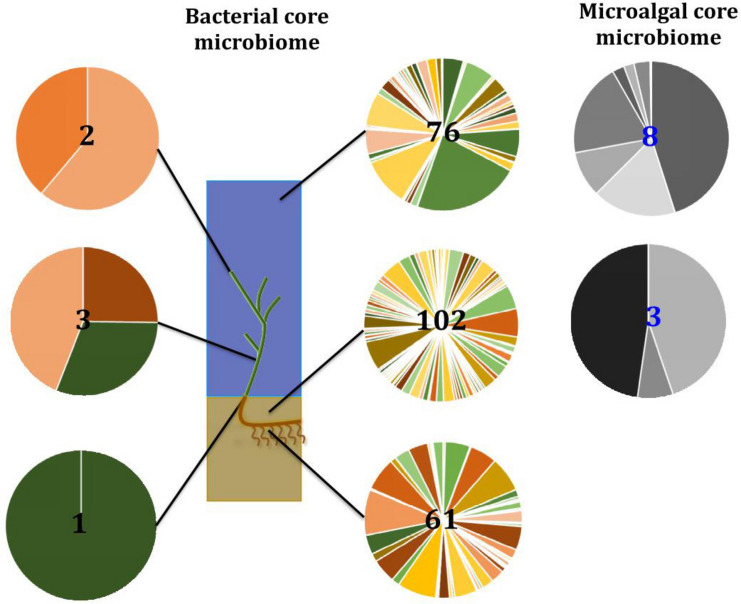
Ecological dynamics of the seagrass microbiomes. Distinct microbial communities live in association with disparate sections of the plant (i.e., upper leaf, lower leaf, sheath, and roots and rhizomes) and its surroundings (i.e., surficial sediment and adjacent seawater). Their composition and structure are strongly shaped by the varying conditions offered within each microenvironment and also influenced by the environment. Therefore, specific bacterial (full color) and microalgal (gray) members make up core microbiomes that are different from each other and constitute up to 4% of the entire microbiome. This variability at the microscale is well conserved within each site, and despite the biogeographical changes of microbial communities, there are some microorganisms that consistently occur within microenvironment types. Numbers in the middle represent total number of core members.

Our results indicate that the seagrass species *Z. muelleri* harbors specific microbial assemblages that differ significantly from the adjacent seawater and sediments. Our data also indicate that discrete bacterial, microalgal, and fungal communities occur within specific key seagrass microenvironments, and that the identity of members of these microenvironment-specific communities are often conserved across geographically disparate habitats (Figure 13). Indeed, for all three microbial taxa, differences in community composition between the specific seagrass microenvironments, which were generally separated by just a few centimeters, were significantly greater than the differences observed between geographical locations spanning 86 km of coastline. These results indicate, that as with many other organisms, seagrasses host several discrete microbial assemblages that are each adapted to local environmental conditions.

In the case of bacteria, for example, members of the Pseudomonadaceae, Rhodobacteraceae, and Comamonadaceae are dominant features of the microbiome inhabiting the *Z. muelleri* phyllosphere, where they exploit the oxic conditions and high levels of dissolved organic carbon on the leaf surface (Hirano and Upper, 2000; Dixon and Kahn, 2004; Haselkorn and Kapatral, 2005; Jorgensen et al., 2009; Juárez-Jiménez et al., 2010). On the other hand, sulfate reducing and sulfur oxidizing bacteria from the Desulfobacteraceae and Chromatiales are dominant core microbiome members within the roots and rhizomes, where they likely regulate the carbon and sulfur cycling processes that influence the decomposition of organic material and ultimately the health of the host (Storelli et al., 2013; Kleindienst et al., 2014; Thomas et al., 2014; Varon-Lopez et al., 2014; Lehnen et al., 2016).

Overall, our study demonstrates that while the seagrass microbiome is highly heterogeneous at small-scales, specific microbial assemblages are organized according to local environmental conditions, with this structure maintained across broad geographic scales. These patterns are indicative of highly specialized, and likely ecologically important, roles of the seagrass microbiome, with bacterial, microalgal and fungal assemblages shifting according to the changing conditions across the disparate microhabitats within the plant and its surroundings. Our findings provide fundamental, baseline information of the composition and structure of microbial communities associated with *Z. muelleri*. Future work defining the seagrass microbiome function by using, for instance, metagenomics approaches will be critical in evaluating the relevance of particular seagrass-microbe associations.

## Author Contributions

VH-M conceived the study, designed the sampling strategy, conducted the fieldwork and lab work, developed the methodological approaches, analyzed the data, drafted the manuscript, prepared the figures and tables and obtained the approval of the final submission. TK supported the bioinformatic analyses, developed the customized pipelines for data analysis, and provided important contributions to the results interpretation. KP assisted on the statistical analyses and provided critical contributions to the results interpretation. TJ participated in fungal sequencing and data analysis. PR supervised the study. JS conceived the study, designed the sampling strategy, provided the regular supervision of VH-M throughout the data analysis and interpretation, drafted the manuscript and substantially contributed to its intellectual content. All authors agreed to be accountable for the content of the work.

## Conflict of Interest

The authors declare that the research was conducted in the absence of any commercial or financial relationships that could be construed as a potential conflict of interest.
