# Corrigendum: Regional and Microenvironmental Scale Characterization of the *Zostera muelleri* Seagrass Microbiome

**DOI:** 10.3389/fmicb.2021.642964

**Published:** 2021-03-10

**Authors:** Valentina Hurtado-McCormick, Tim Kahlke, Katherina Petrou, Thomas Jeffries, Peter J. Ralph, Justin Robert Seymour

**Affiliations:** ^1^Climate Change Cluster, Faculty of Science, University of Technology Sydney, Ultimo, NSW, Australia; ^2^School of Life Sciences, Faculty of Science, University of Technology Sydney, Ultimo, NSW, Australia; ^3^School of Science and Health, Western Sydney University, Penrith, NSW, Australia

**Keywords:** seagrass microbiome, diversity, core, bacteria, microalgae, fungi, amplicon sequencing

In the original manuscript, there was an error with our bioinformatics workflow, whereby seagrass-associated reads were not removed prior to taxonomic profiling of the fungal community in seagrass-associated microenvironments. As a consequence, incorrect assignment of some seagrass ITS sequences as fungal sequences occurred in some instances, leading to inflated levels of fungi, particularly from the Glomeraceae and Rhytismataceae groups. We have re-analyzed the data after ensuring that seagrass ITS sequences have not been incorrectly assigned as fungi. Due to the low number of fungal sequencing reads from the leaf that remained after removal of putative seagrass sequences, only the mycobiomes associated with seagrass roots and rhizomes, sediments and seawater were compared using exactly the same approaches described in the published manuscript. Text from the original article has been updated to reflect a shift in patterns in mycobiome data that resulted from the exclusion of ITS sequences that were, subsequent to the publication of Hurtado-McCormick et al. (2019), found to be associated with seagrass leaf material rather than fungi. As a consequence, this led to the removal of reference to Glomeraceae and Rhytismataceae data as following new analysis it was determined that sequences assigned to these groups are present in lower proportions than originally reported. Corrections have been made to the interested sections.

A correction has been made to **Materials and Methods** section, **Sequence Data Analysis, paragraph 3**:

Initial sequence processing for fungal ITS genes was conducted using QIIME, v1.9.1 (Caporaso et al., 2010). Briefly, low-quality regions were trimmed from the 5′ end of sequences, and paired ends were joined with fastq-join (Aronesty, 2011, 2013) and de-multiplexed. Sequences containing ambiguous bases were removed from the dataset along with low-quality reads and chimeric sequences. Referenced-based chimera detection (Nilsson et al., 2015) was performed using the UCHIME algorithm from the USEARCH package (Edgar, 2010; Edgar et al., 2011) implemented within VSEARCH, v2.3.2 (Rognes et al., 2016). OTUs were defined as clusters of 97% sequence similarity using UCLUST (Edgar, 2010). The resultant OTU table was filtered to remove singletons and seagrass-affiliated sequences. OTU sequences were screened for non-fungal sequences using BLAST (Altschul et al., 1990), against the nucleotide database from the National Center for Biotechnology Information (NCBI). Non-fungal sequences were identified using BASTA (Kahlke and Ralph, 2019) and the following parameters: -l 250 (sequence length), -m 0 (mismatches), and -i 97 (identity). These sequences were subsequently removed from the dataset. Final taxonomies were assigned to the filtered OTU set (i.e., sequences of unknown origin) using the UNITE database v6.9.7 (Koljalg et al., 2013), BLAST, and vBLASTC (Altschul et al., 1990). Finally, the resultant filtered OTU table was rarefied to an even number of sequences per samples to ensure equal sampling depth (i.e., lower number of sequences per sample = 1456). Given the nature of this study's experimental design and the importance of replication in complex datasets, the rarefaction cut-off was chosen to include at least triplicates per sample type (**Supplementary Table 9**). Due to the low number of fungal sequencing reads from the leaf that remained after removal of putative seagrass sequences, only the mycobiomes associated with seagrass roots and rhizomes, sediments and seawater were used for further post-sequencing analyses, while the seagrass leaf samples were omitted from cross-sample comparisons. A separate re-analysis of these samples with unrarefied data supported all of the scientific conclusions of our original manuscript, except for the predominance of the Rhytismataceae family in the upper leaf mycobiome, which instead represented rare taxa (relative abundance <1% in all samples) within seagrass-associated fungal communities.

A correction has been made to **the Results and Discussion** section, **The Seagrass Mycobiome, paragraphs 1-3** and **5:**

Although less studied in seagrasses, several fungi have been demonstrated to be highly beneficial for aquatic and terrestrial plant fitness while establishing intimate relationships with their host (i.e., mycorrhizal associations) to facilitate nutrient uptake or compete against other potentially pathogenic microbes (Azcon-Aguilar et al., 1999; Kohout et al., 2012; Raghukumar, 2012). In this study, fungal communities associated with *Z. muelleri* displayed significantly different levels of alpha diversity for the two measured indices (Chao1 and Shannon's Index) between both seagrass microenvironments (*p*_Chao1_ = 0.0006, $p_{\text{Shanno}{\text{n}}^{\text{′}}\text{s}}$ = 0.0001) and sampling sites (*p*_Chao1_ = 0.0051, $p_{\text{Shanno}{\text{n}}^{\text{′}}\text{s}}$ = 0.0010) (Supplementary Table 7). Our *post-hoc* analyses indicated that the differences across habitats were mostly driven by differences between Lake Macquarie and all other sites. At Lake Macquarie, fungal microbiomes exhibited significantly lower levels of alpha diversity (*p* < 0.05, Supplementary Table 7). Moreover, several significant differences in alpha diversity were observed between seagrass microenvironments within each site (Supplementary Table 7). Even though some of these differences varied from site to site, general patterns were similar to those observed for microalgal assemblages, whereby seagrass-associated microenvironments (here roots and rhizomes only) had lower levels of fungal diversity than the surrounding seawater and sediments (Figure 2). This is possibly due to antifungal chemical defenses and physiological responses from the host against co-occurring marine fungi, which have been well described for other seagrass species (Ross et al., 2008).

Consistent with the patterns observed for bacterial and microalgal assemblages, fungal community structure varied significantly across both seagrass microenvironments (*p* = 0.0001) and sampling sites (*p* = 0.0001). Notably, all sites differed significantly from each other (*p* < 0.05). However, the differences between microenvironments within each sampling location explained a greater level of variation between mycobiomes compared to the differences between sites [ECV, Mi(Si) = 1262.70, Supplementary Table 8]. Roots and rhizomes, sediment and seawater communities formed discrete, separated clusters within each site, as evidenced in nMDS (Figure 10) and CLUSTER (Supplementary Figure 7) analyses. However, such clear separation of fungal communities between microenvironments was not apparent in unrarefied data from leaf samples (Supplementary Figure 6).

Fungal OTUs identified within four taxonomic groups consistently dominated fungal assemblages across the three microenvironments and four sampling locations studied here (Figures 11, 12). This is consistent with the hypothesis of extreme ecological flexibility acclaimed for obligate marine fungal species (Nicoletti and Andolfi, 2018). OTUs matching the order Pleosporales (291 unique OTUs) and the species *Wallemia ichthyophaga* (54 unique OTUs) represented the most abundant fungi across the roots and rhizomes, sediments and seawater microenvironments, making up an average of 38 and 18% of these communities, respectively (Figures 11, 12). Many freshwater and marine species of Pleosporales have been described to date, including several endophytes and saprophytes of plants, as well as symbionts, parasites and pathogens of seagrasses and marine macroalgae (Suetrong et al., 2009; Zhang et al., 2009; Boonmee et al., 2012; Hyde et al., 2013; Hashimoto et al., 2017). Some species are also dominant members of microbiomes associated with mangroves, showing a microenvironmental preference for intertidal parts of the host, which occur above the water level (Raghukumar, 2012). Our observations of predominant Pleosporales OTUs across all three microenvironments and particularly within roots and rhizomes, where these fungi represented 55% of the mycobiome, are highly consistent with previous reports of the dominance of a single marine fungus from the Pleosporales, probably representing a new genus, associated with the roots of the seagrass species *Posidonia oceanica* (Vohník et al., 2016). While, to our knowledge, the other dominant fungal species, *W. ichthyophaga*, has not previously been reported in seagrasses, it has been found to occur in association with other benthic marine organisms, including corals (Raghukumar, 2012). We also observed OTUs that dominated the microenvironments surrounding the seagrass. These included the species *Mortierella horticola* (42 unique OTUs) and unclassified members of the Pezizomycetes class (7 unique OTUs), which represented 7 and 0.38% of the sediment and seawater fungal communities, respectively (Figures 11, 12). Despite its low relative abundance, the Pezizomycetes was the only taxon that differed significantly across the three seagrass microenvironments (*p* = 0.013, Figure 12). Nevertheless, further exploration of our beta diversity data revealed high relative abundances and high contributions to microenvironmental dissimilarities of the other fungi aforementioned, suggesting their potential importance within the seagrass mycobiome.

Here we chose to use 97% similarity criteria for defining fungal OTUs characterized using our ITS sequencing approach, which we consider a suitable conservative approach given the lower levels of taxonomic diversity covered in fungal ITS databases (relative to e.g., bacteria) and is consistent with values previously used to characterize the mycobiome associated with terrestrial plants (Giordano et al., 2009) and coastal grasses (Sánchez-Márquez et al., 2008). The overall dominance of four taxonomic groups across the three microenvironments studied here is in line with previous observations of very narrow mycobiomes associated with seagrasses (Devarajan and Suryanarayanan, 2002; Vohník et al., 2016), plants from salt marshes (Al-Nasrawi and Hughes, 2012), mangroves (Xing and Guo, 2011), and other aquatic plants (Kohout et al., 2012). Nevertheless, and similar to microalgae, we did not observe a conserved “core” of fungal associates within any of the seagrass microenvironments (here roots and rhizomes only), and a core fungal microbiome of two members was only identified for the seawater microenvironment. These core OTUs belonged to the family Glomeraceae and the order Pleosporales. Our results are indicative of a weaker ecological coupling between seagrasses and fungal taxa, relative to that observed for seagrass bacterial interactions. We propose that, relative to bacteria, which appear to display highly specific interactions with different components of the plant due to a stronger influence of the conditions at the microscale, seagrass-associated fungi appear to establish more generalist relationships with their host.

In the original article, there was a mistake in some figures and tables.

The corrected Figure 2 appears below. Secondary Y-axis titles have been added to the figure to improve clarity. The figure legend was not interested and has not changed.

**Figure 2:**
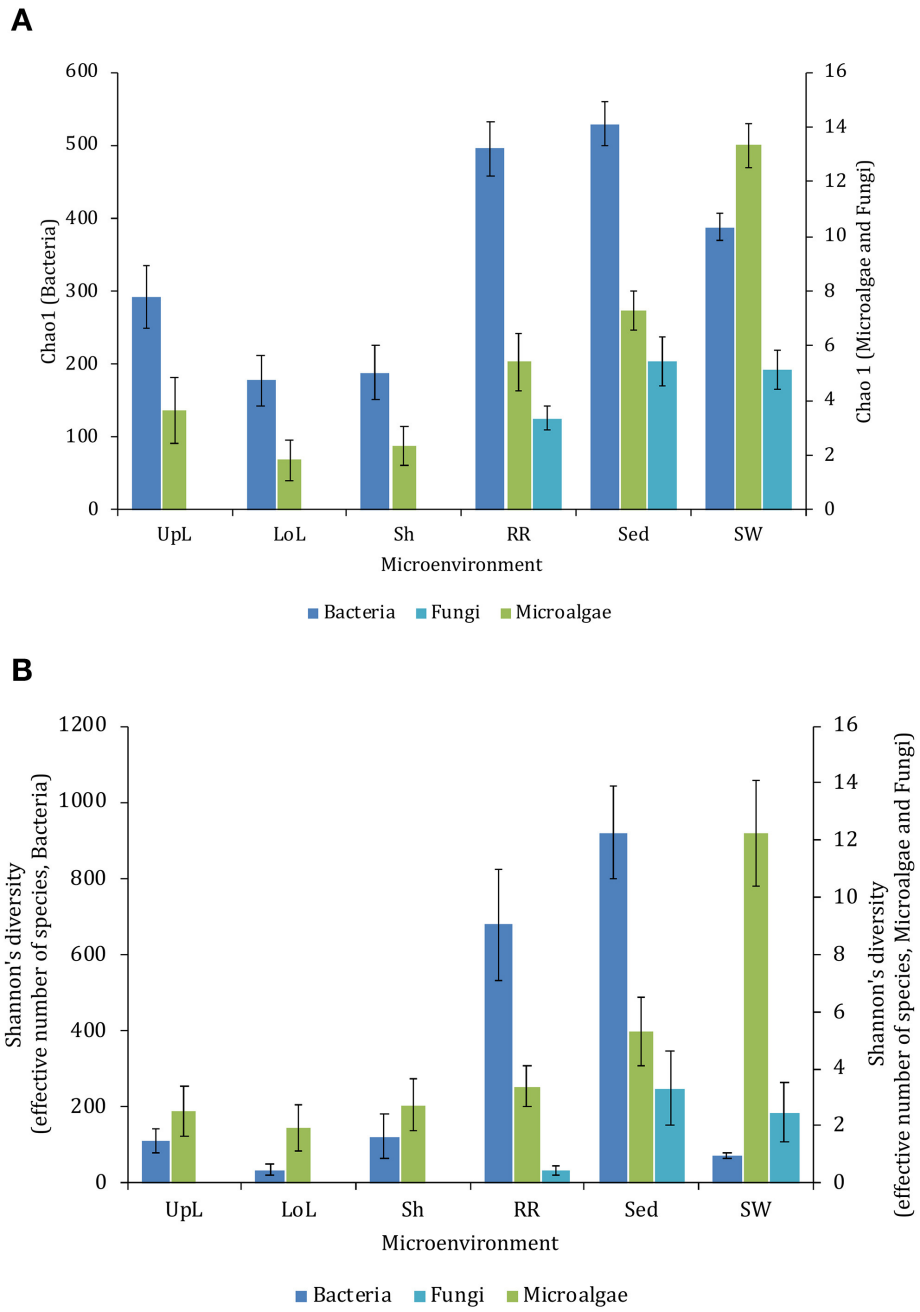
Microbial mean alpha diversity across seagrass microenvironments. Multiple comparisons between Chao1 diversity **(A)** and Shannon's diversity index **(B)**, calculated for each taxa and microenvironment separately, were tested for statistical significance with Permutational Multivariate Analysis of Variance (PERMANOVA, Minkowski metric distance matrix and nested design). Mean values for each microenvironment are shown, and error bars reflect the standard error of the mean.

The corrected Figure 10 and Figure 10 legend appear below.

**Figure 10:**
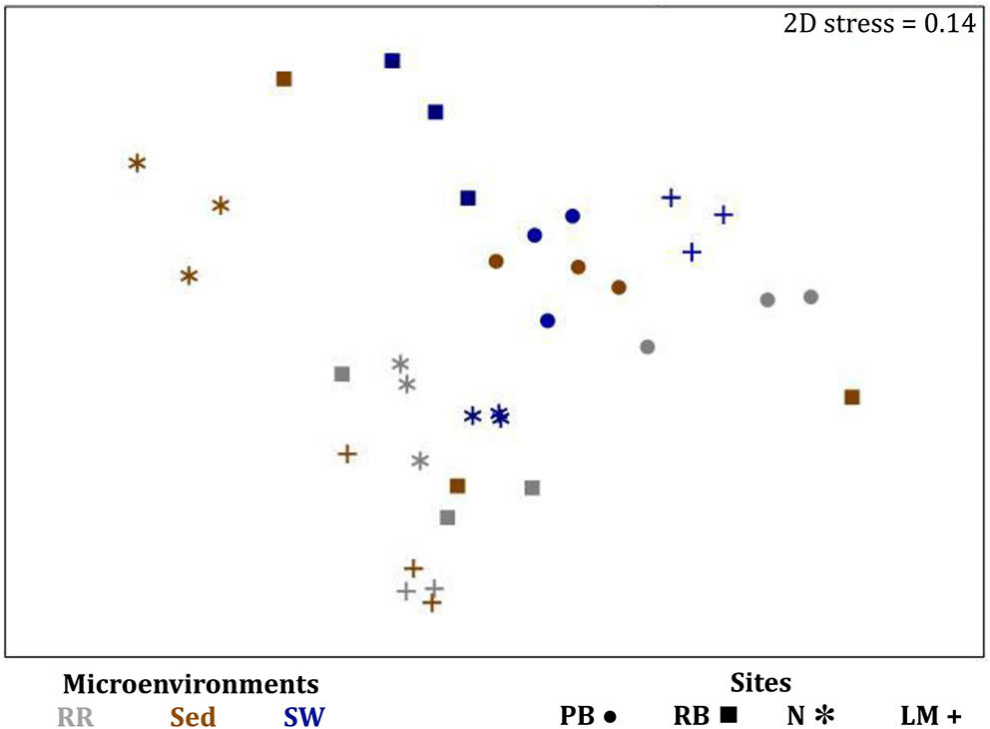
Microenvironmental and regional partitioning of the seagrass fungal microbiome. Non-parametric multidimensional scaling (nMDS) of fungal microbiomes (*n* = 35), based on a lower triangular resemblance calculated with the S17 Bray–Curtis similarity measure from relative abundances of OTUs (high values down-weighted with square root). Samples are colored by microenvironment (RR, roots and rhizomes; Sed, sediment, SW, seawater), with different shapes for sites (PB, Palm Beach; RB, Rose Bay; N, Narrabeen Lagoon; LM, Lake Macquarie). Sample clustering patterns by microenvironment within each site represent the level of similarity between samples based on the degree to which OTUs are shared between them. The 2D stress is shown in the upper right corner of the nMDS plot (Kruskal stress formula = 1, minimum stress = 0.01). The nMDS for the three microenvironments associated with the leaf is provided in Supplementary Figure 6 and a hierarchical cluster analysis (CLUSTER) is provided in Supplementary Figure 7.

The corrected Figure 11 and Figure 11 legend appear below.

**Figure 11:**
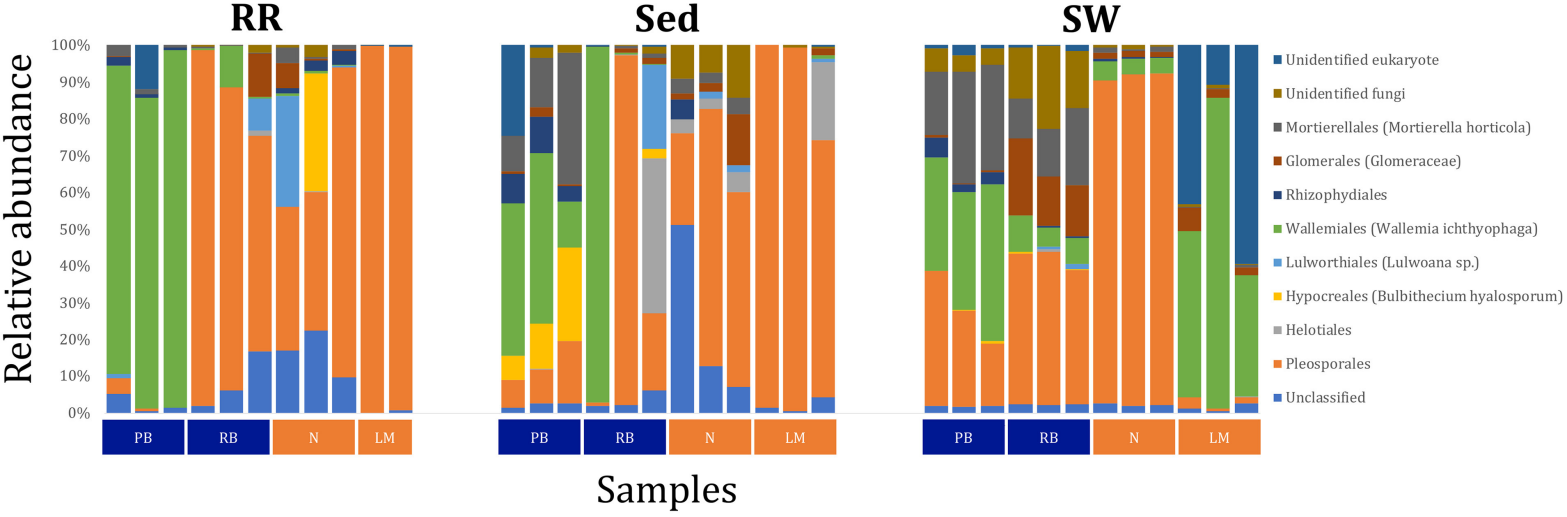
Fungal community composition across seagrass microenvironments. Beta diversity of fungal microbiomes across the three seagrass microenvironments studied here. Triplicate samples per microenvironment within each of the four sampling sites (*n* = 35) are colored by the highest assigned taxonomic level. Unique OTUs were summarized at the species level, and the representation of taxonomic groups within each sample are plotted. Only representative species with a relative abundance > 1% in all samples are shown to help remove visual clutter. RR, roots and rhizomes; Sed, sediment; SW, seawater; PB, Palm Beach; RB, Rose Bay; N, Narrabeen Lagoon; LM, Lake Macquarie.

The corrected Supplementary Table 8 appears below.

**Supplementary Table 8:**
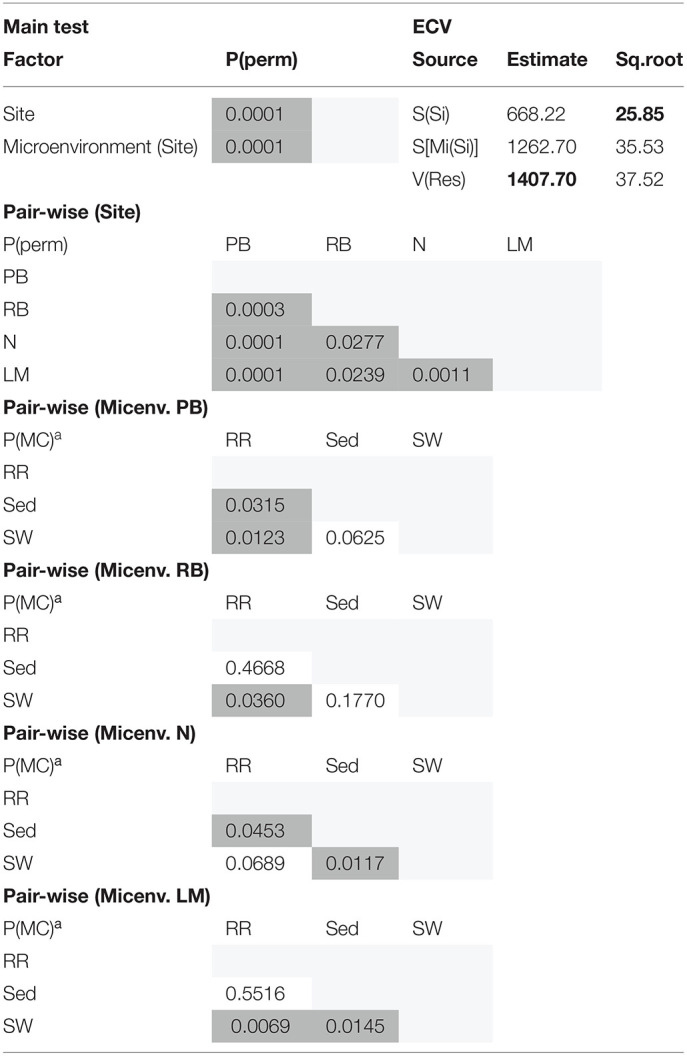
Statistical analyses for multidimensional scaling (Fungi).

*Differences between fungal communities across sites and microenvironments (top), between sites (middle), and between microenvironments within sites (bottom) were tested for statistical significance in Permutational Multivariate Analysis of Variance (PERMANOVA, Bray-Curtis dissimilarity matrix, nesteddesign)*.

*^a^Monte Carlo p-values used when there were not enough possible permutations (unrestricted permutation method, 9,999permutations)*.

*Significant values at the 0.05 level are shown ingrey*.

*ECV, estimates of components of variation; RR, roots and rhizomes; Sed, sediment; SW, seawater; PB, Palm Beach; RB, Rose Bay;N, Narrabeen Lagoon; LM, Lake Macquarie*.

The corrected Figure 12 and Figure 12 legend appear below.

**Figure 12:**
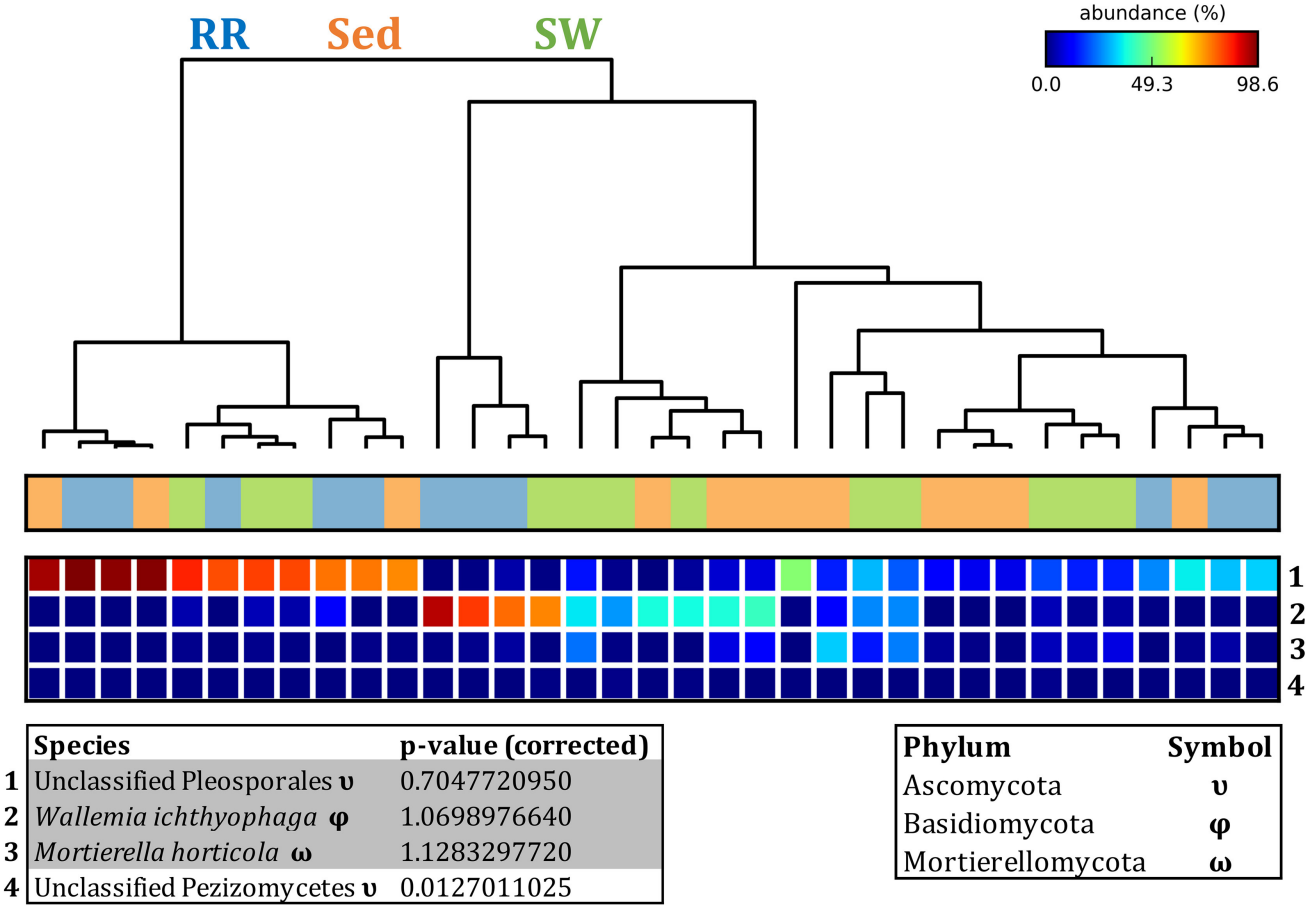
Fungal discriminatory OTUs at the microenvironmental scale. Extensive hypothesis testing of taxonomic profiles was coupled with similarity percentages analyses (SIMPER) for fungal microbiomes across microenvironments. The proportion of sequences (mean frequency %) of OTUs significantly over-represented (Kruskal–Wallis H-test, α = 0.05, effect sizes: η^2^) and consistently contributing to the differences between microenvironments is indicated by varying color intensities. Corrected *p*-values were calculated using the Benjamini–Hochberg's approach. Two-way crossed SIMPER analyses were performed with site and microenvironment variables as factors (S17 Bray–Curtis similarity matrix). High contributors were selected from the top-5 contributors of each pair-wise comparison between microenvironments, and those OTUs consistently accounting for the dissimilarities between any given microenvironment and the other two microenvironments were chosen as high contributors to couple with the statistical results. High contributors that were significantly over-represented were classified as discriminatory OTUs (i.e., 1–4). OTUs are sorted by decreasing mean abundance, and samples are clustered by average neighbor distance (UPGMA, distance threshold = 0.75) and colored by microenvironment. Different symbols represent the distribution of enriched phyla. High contributors that were not significantly over-represented (gray) were also classified as discriminatory OTUs if their contribution to the differences between microenvironments was consistent. RR, roots and rhizomes; Sed, sediment; SW, seawater.

The corrected Supplementary Figure 6 and Supplementary Figure 6 legend appear below.

**Supplementary Figure 6:**
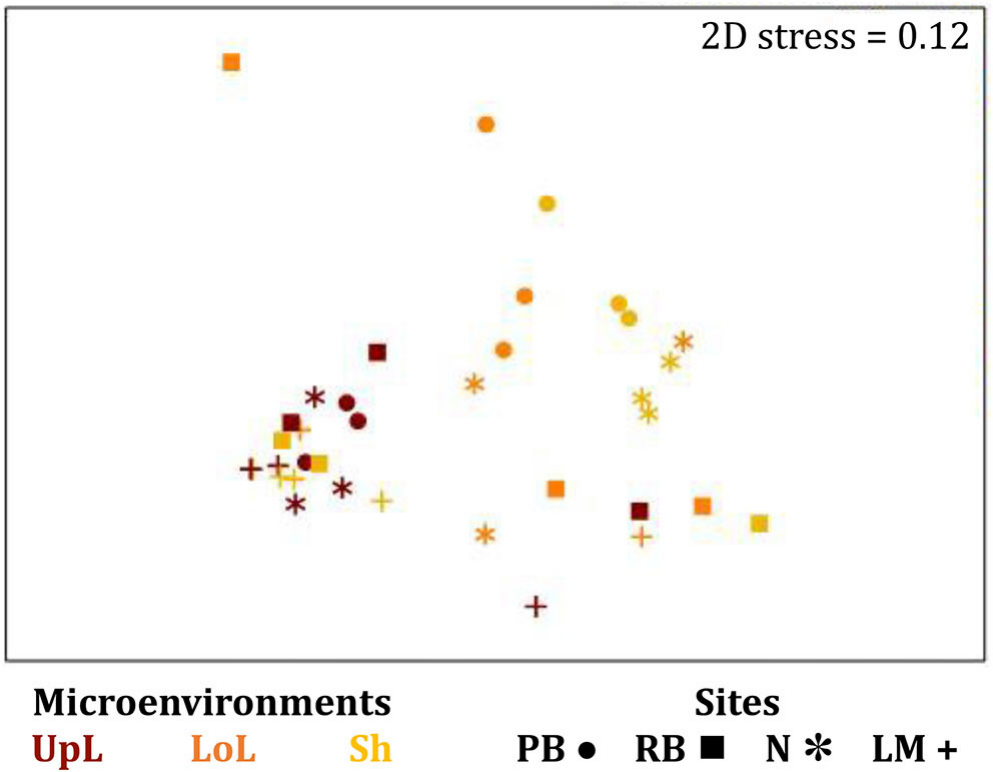
The seagrass leaf-associated microbiomes (Fungi, unrarefied data). Non-parametric multidimensional scaling (nMDS) of fungal microbiomes associated with the seagrass leaf (*n* = 36), based on a lower triangular resemblance calculated with the S17 Bray–Curtis similarity measure from relative abundances of OTUs (high values down-weighted with square root). Samples are colored by microenvironment (UpL, upper leaf; LoL, lower leaf; Sh, sheath), with different shapes for sites (PB, Palm Beach; RB, Rose Bay; N, Narrabeen Lagoon; LM, Lake Macquarie). Clustering patterns by microenvironment within each site represent the level of similarity between samples based on the degree to which OTUs are shared between them. The 2D stress is shown in the upper right corner of the nMDS plot (Kruskal stress formula = 1, minimum stress = 0.01).

The corrected Supplementary Figure 7 and Supplementary Figure 7 legend appear below.

**Supplementary Figure 7:**
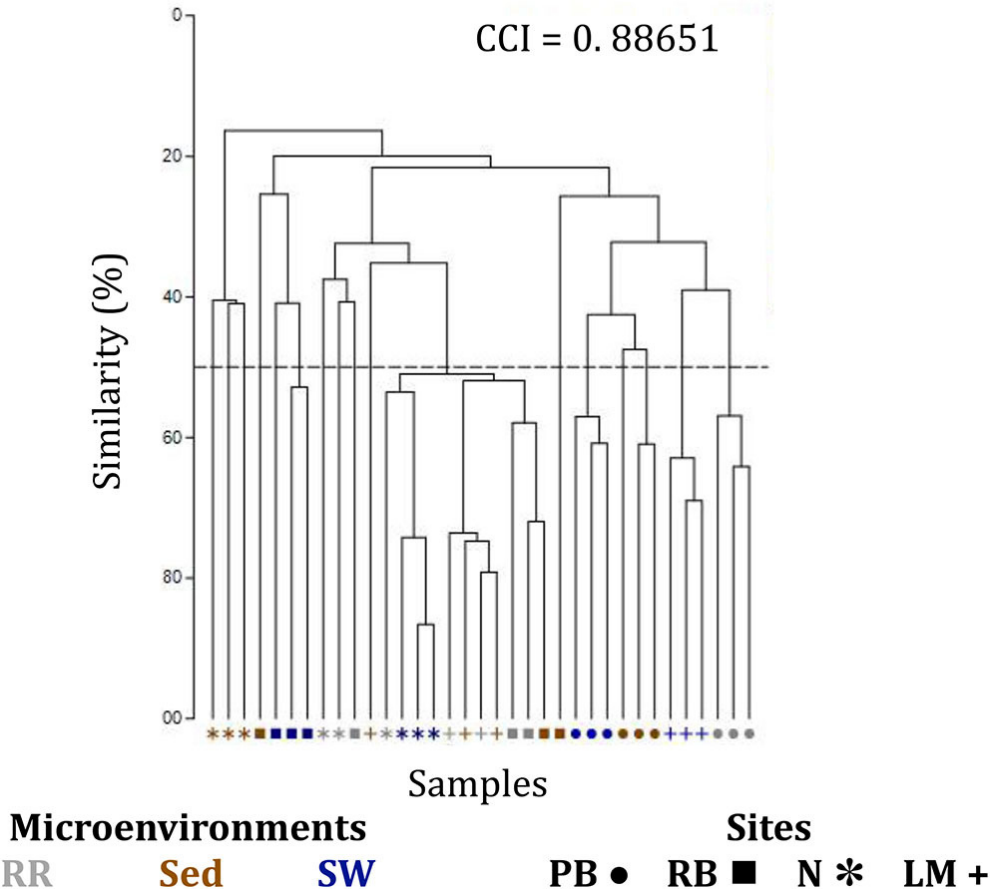
Clustering patterns of the seagrass fungal microbiome. Hierarchical cluster analysis (CLUSTER) of fungal microbiomes, based on a lower triangular resemblance calculated with the S17 Bray-Curtis similarity measure from relative abundances of OTUs (high values down-weighted with square root). Samples are colored by microenvironment (RR, roots and rhizomes; Sed, sediment, SW, seawater), with different shapes for sites (PB, Palm Beach; RB, Rose Bay; N, Narrabeen Lagoon; LM, Lake Macquarie). Group average linkage was used to calculate distances between clusters and generate a dendogram and a cophenetic distance matrix (unsupervised learning method). The cophenetic correlation index (CCI) is provided in the upper right corner of the dendogram, and it was used to assess the faithfulness of the dendogram by computing cophenetic correlation coefficients.

The corrected Supplementary Table 7 appears below.

**Supplementary Table 7:**
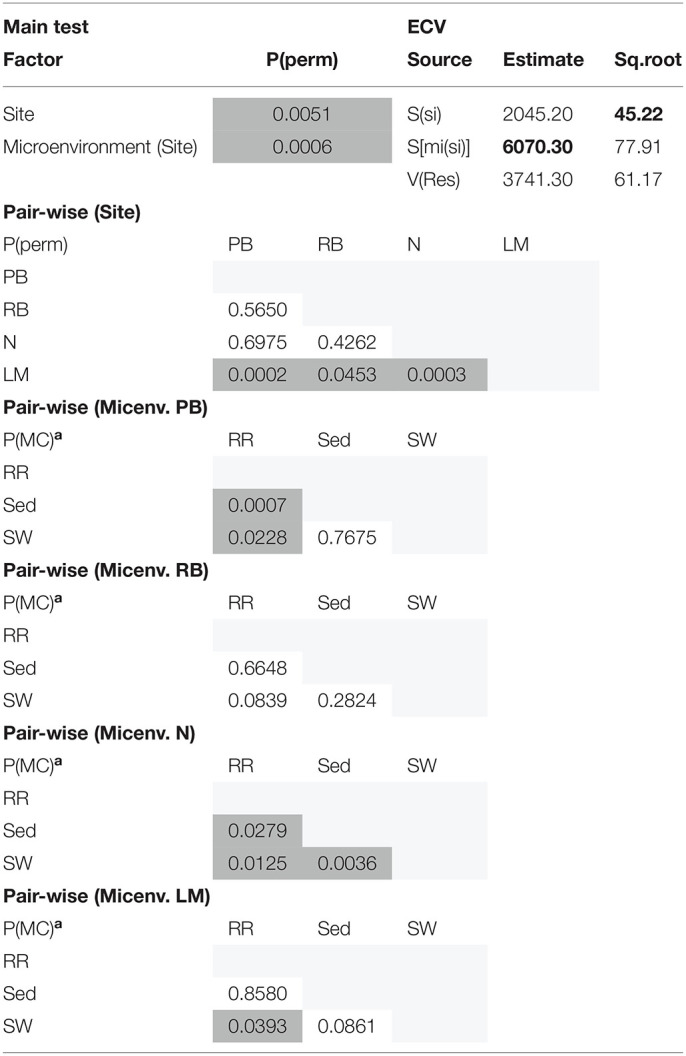
Statistical analyses for fungal mean alpha diversity (Chao1 diversity).

**Supplementary Table 7:**
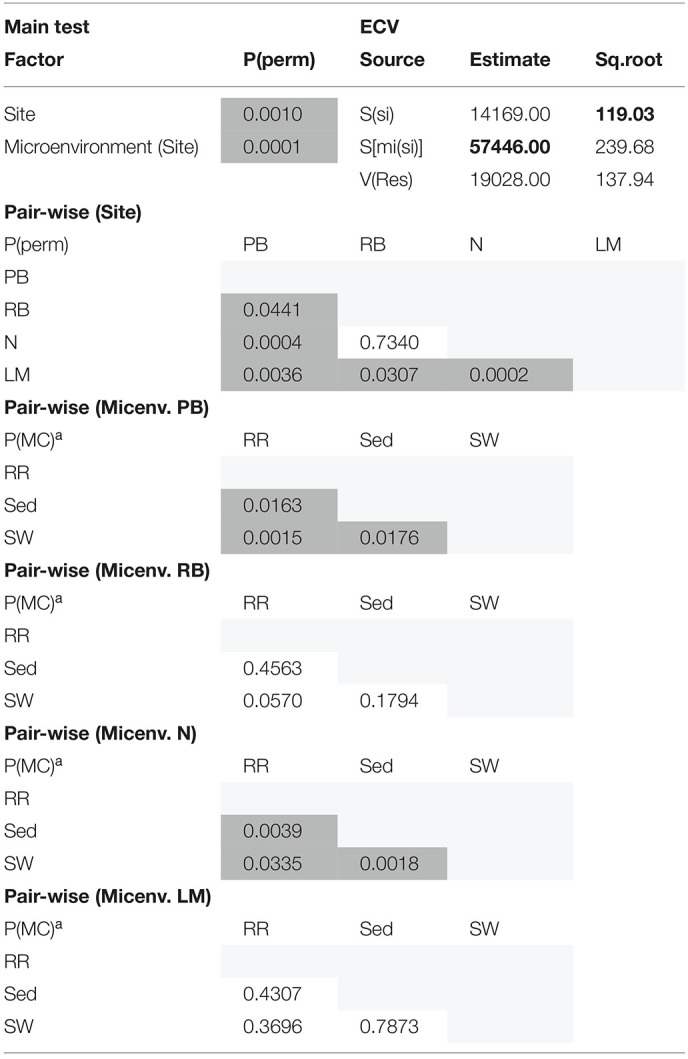
Statistical analyses for bacterial mean alpha diversity (Shannon's diversity index).

*Differences in alpha diversity as measured by Chao1 diversity across sites and microenvironments (main test), between sites and between microenvironments within sites (pair-wise) were tested for statistical significance in Permutational Multivariate Analysis of Variance (PERMANOVA, Minkowski metric distance matrix, nesteddesign)*.

*^a^Monte Carlo p-values used when there were not enough possible permutations (unrestricted permutation method, 9,999permutations)*.

*Significant values at the 0.05 level are shown ingrey*.

*ECV, estimates of components of variation; RR, roots and rhizomes; Sed, sediment; SW, seawater; PB, Palm Beach; RB, Rose Bay; N, Narrabeen Lagoon; LM, LakeMacquarie*.

The authors apologize for this error and state that this does not change the scientific conclusions of the article in any way. The original article has been updated.
