# Supplementary figures and images for: Regional and Microenvironmental Scale Characterization of the Zostera muelleri Seagrass Microbiome
Source: Front Microbiol. 2019 May 14;10:1011. doi: 10.3389/fmicb.2019.01011 (PMC6527750; doi:10.3389/fmicb.2019.01011)

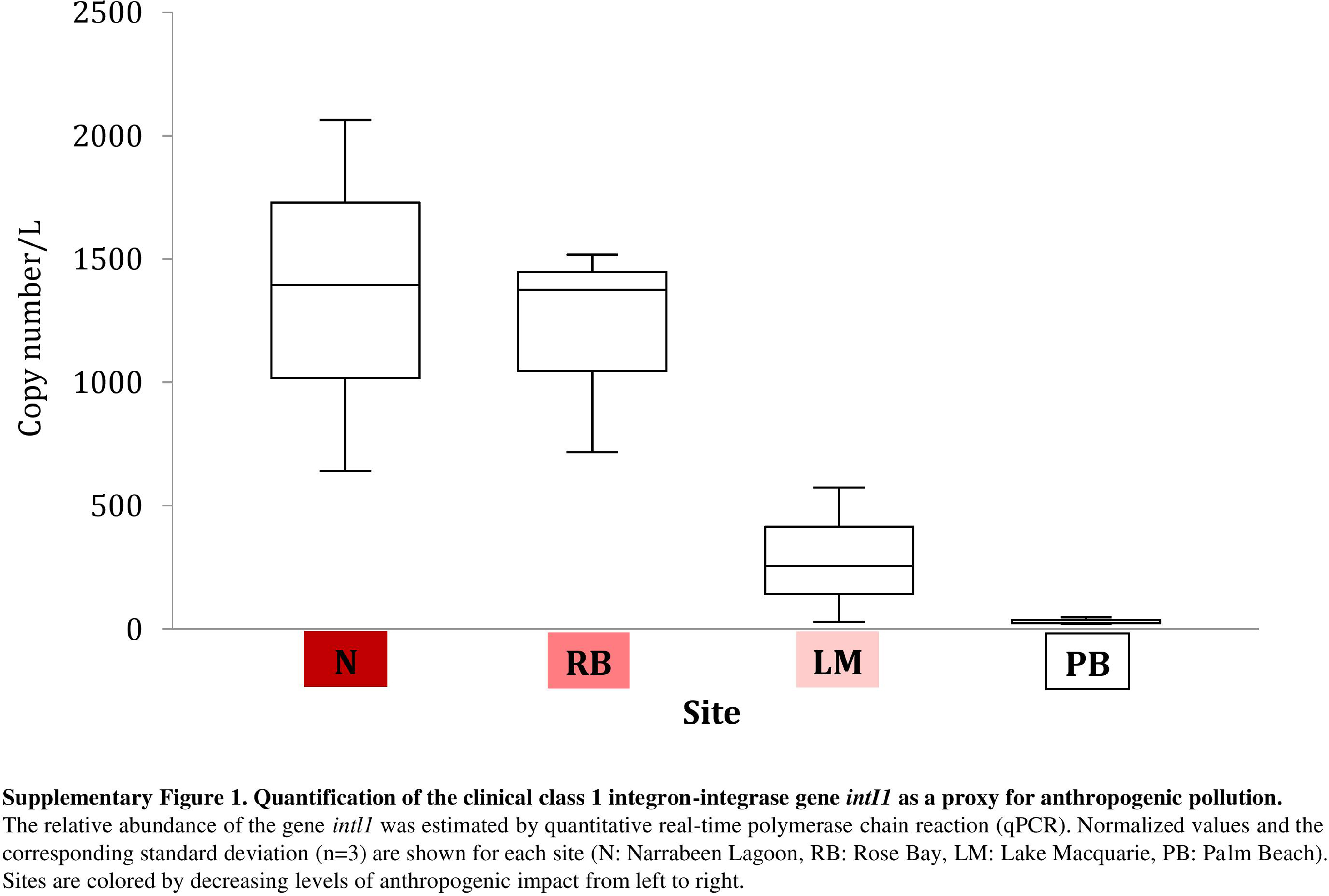

Supplement: Supplementary file 1 [file Image_1.JPEG]

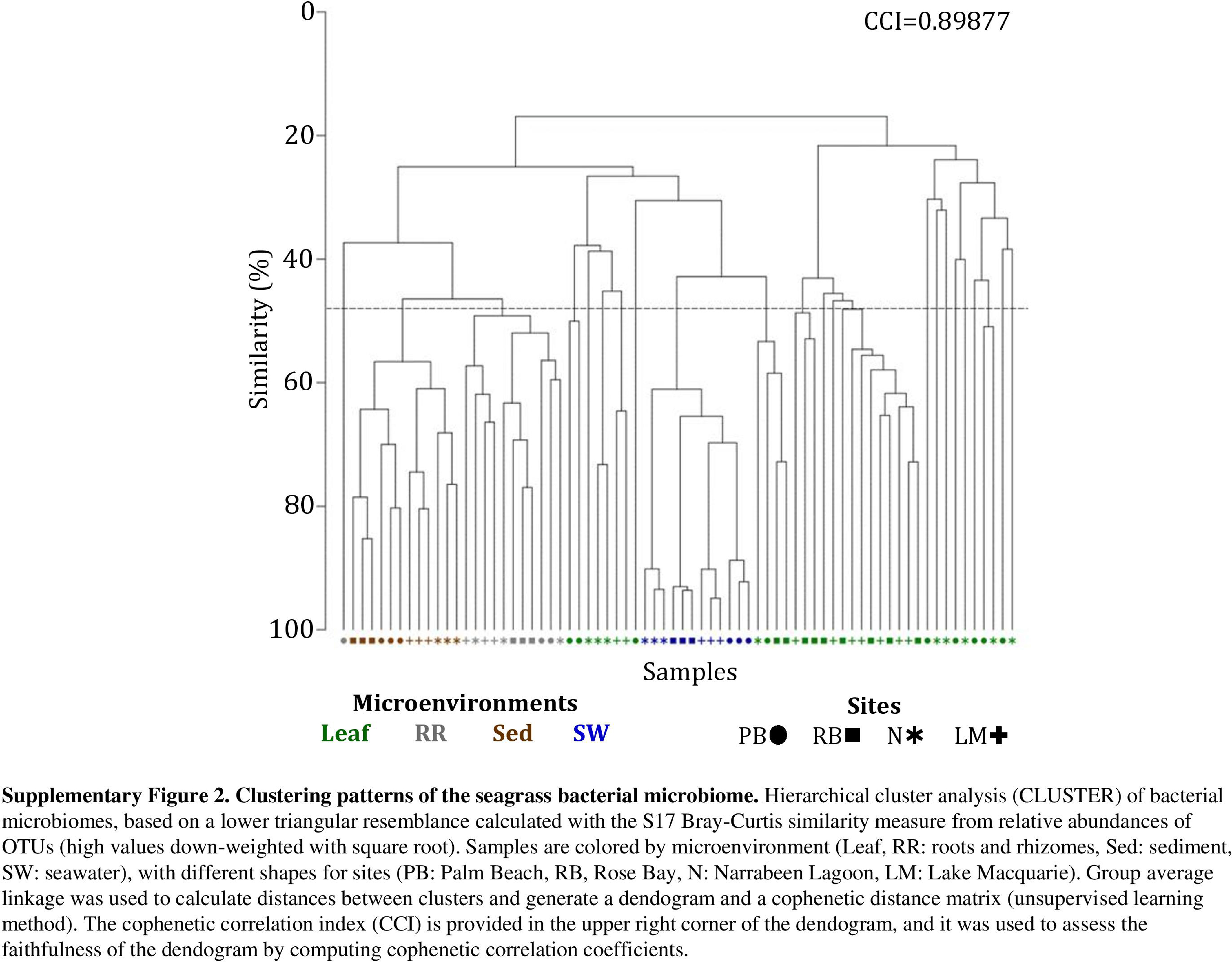

Supplement: Supplementary file 2 [file Image_2.JPEG]

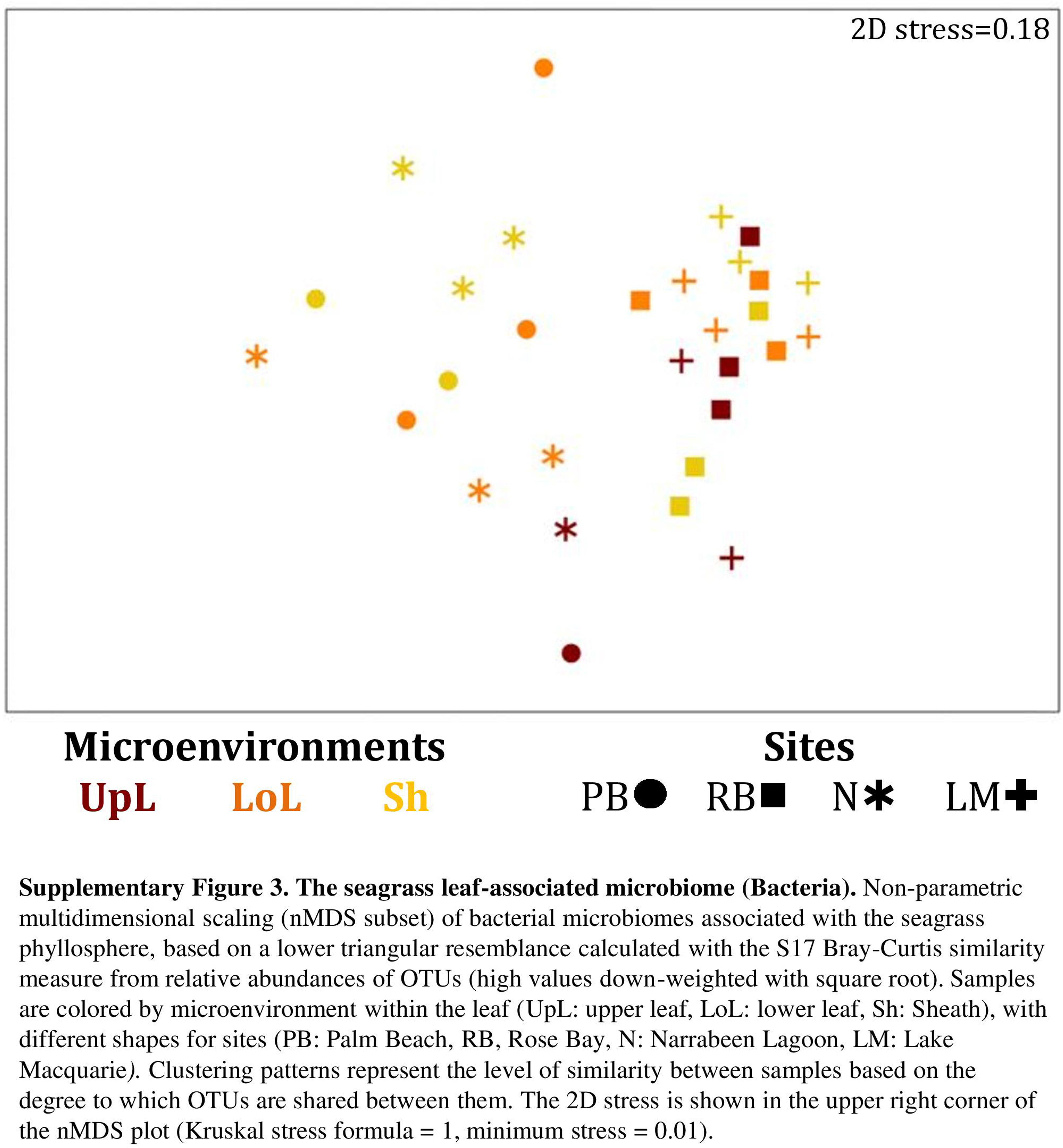

Supplement: Supplementary file 3 [file Image_3.JPEG]

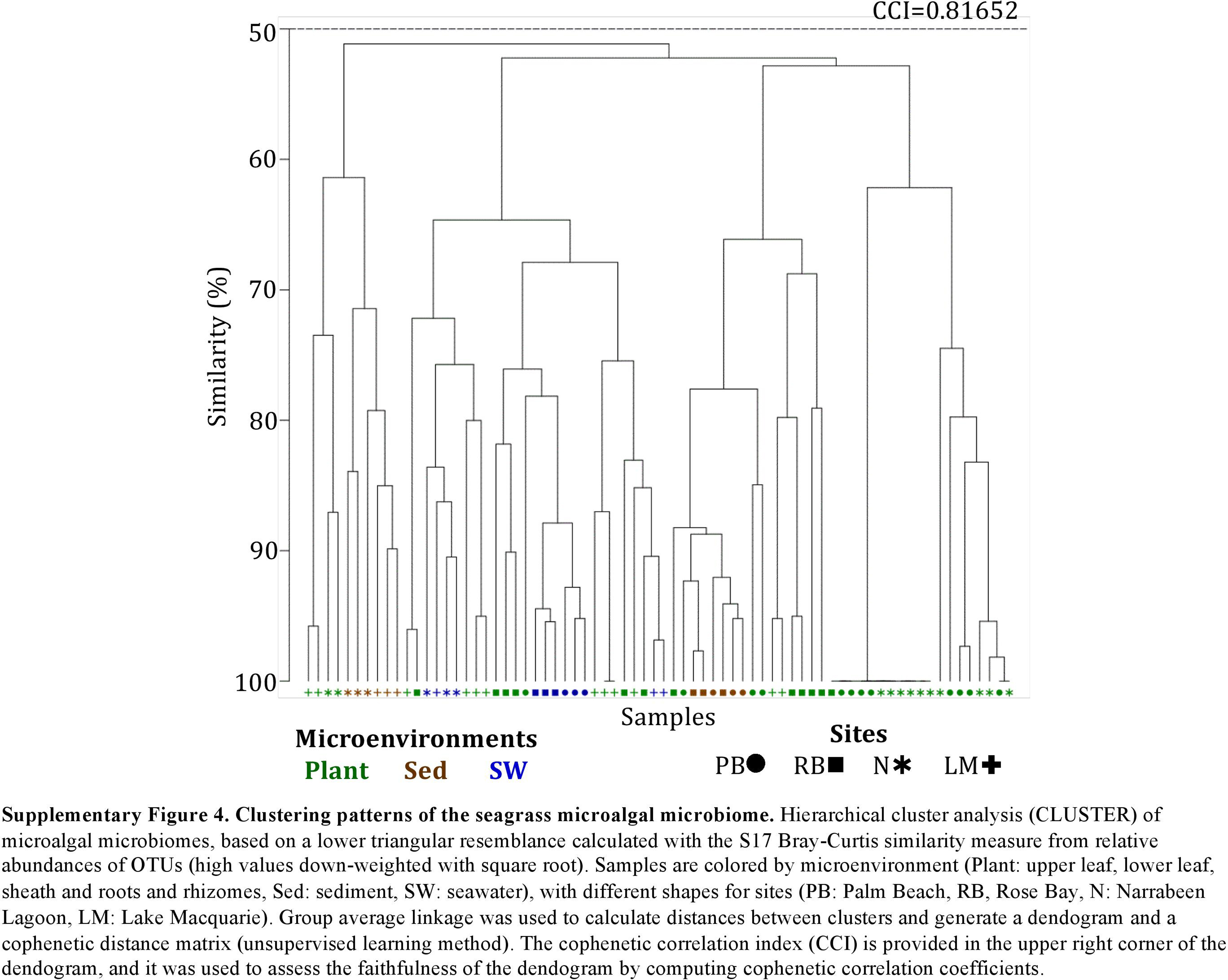

Supplement: Supplementary file 4 [file Image_4.JPEG]

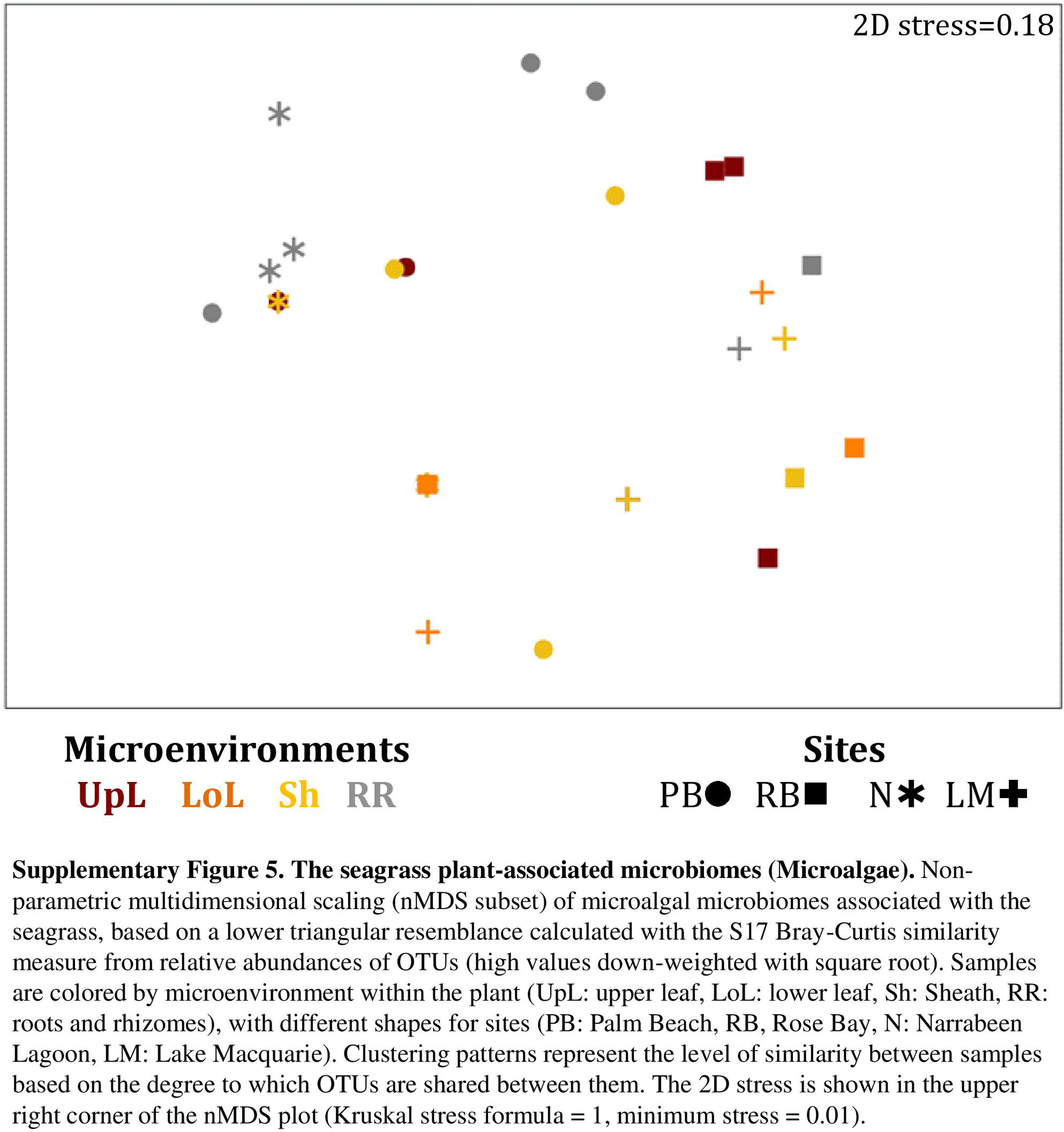

Supplement: Supplementary file 5 [file Image_5.JPEG]

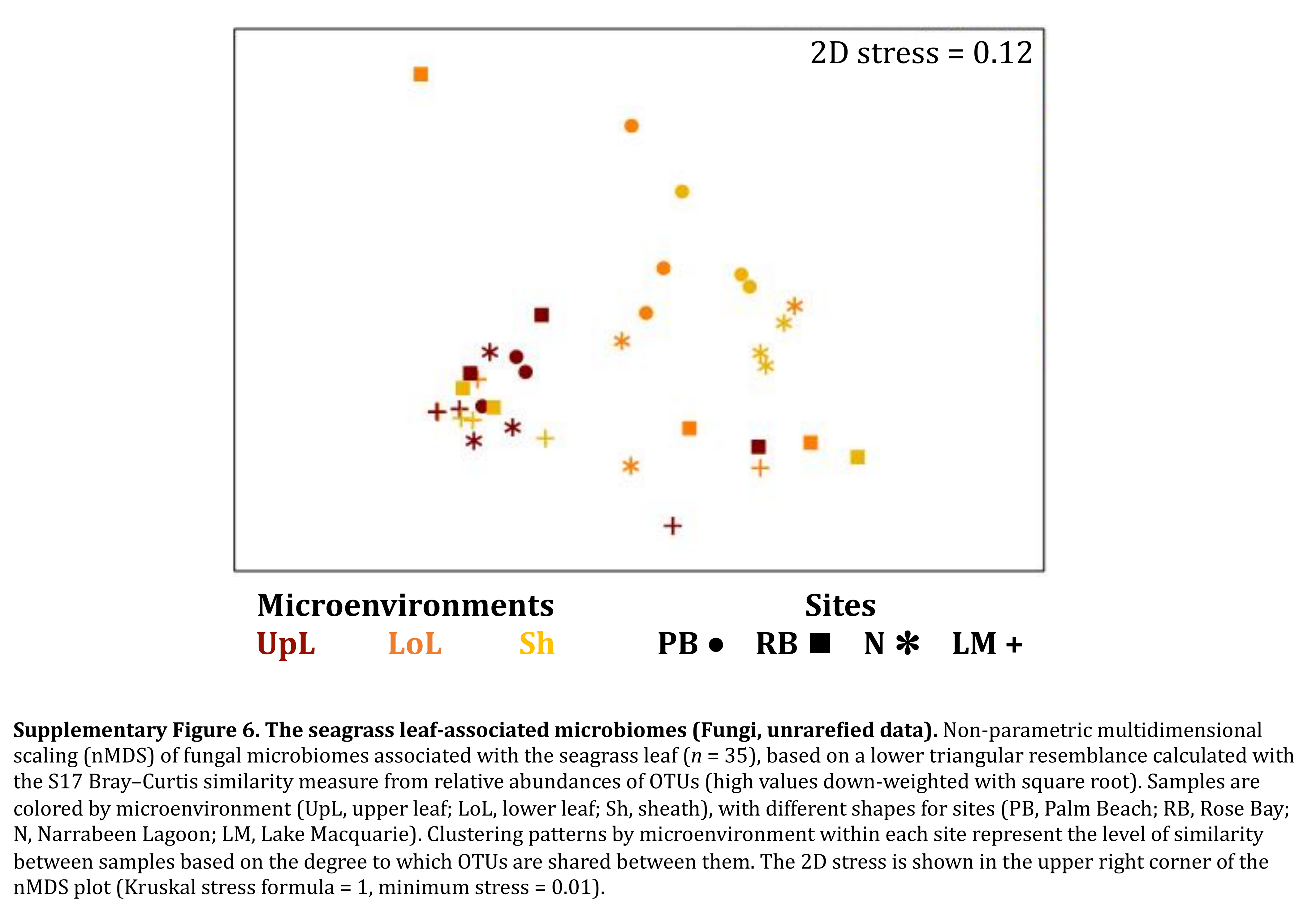

Supplement: Supplementary file 6 [file Image_6.JPEG]

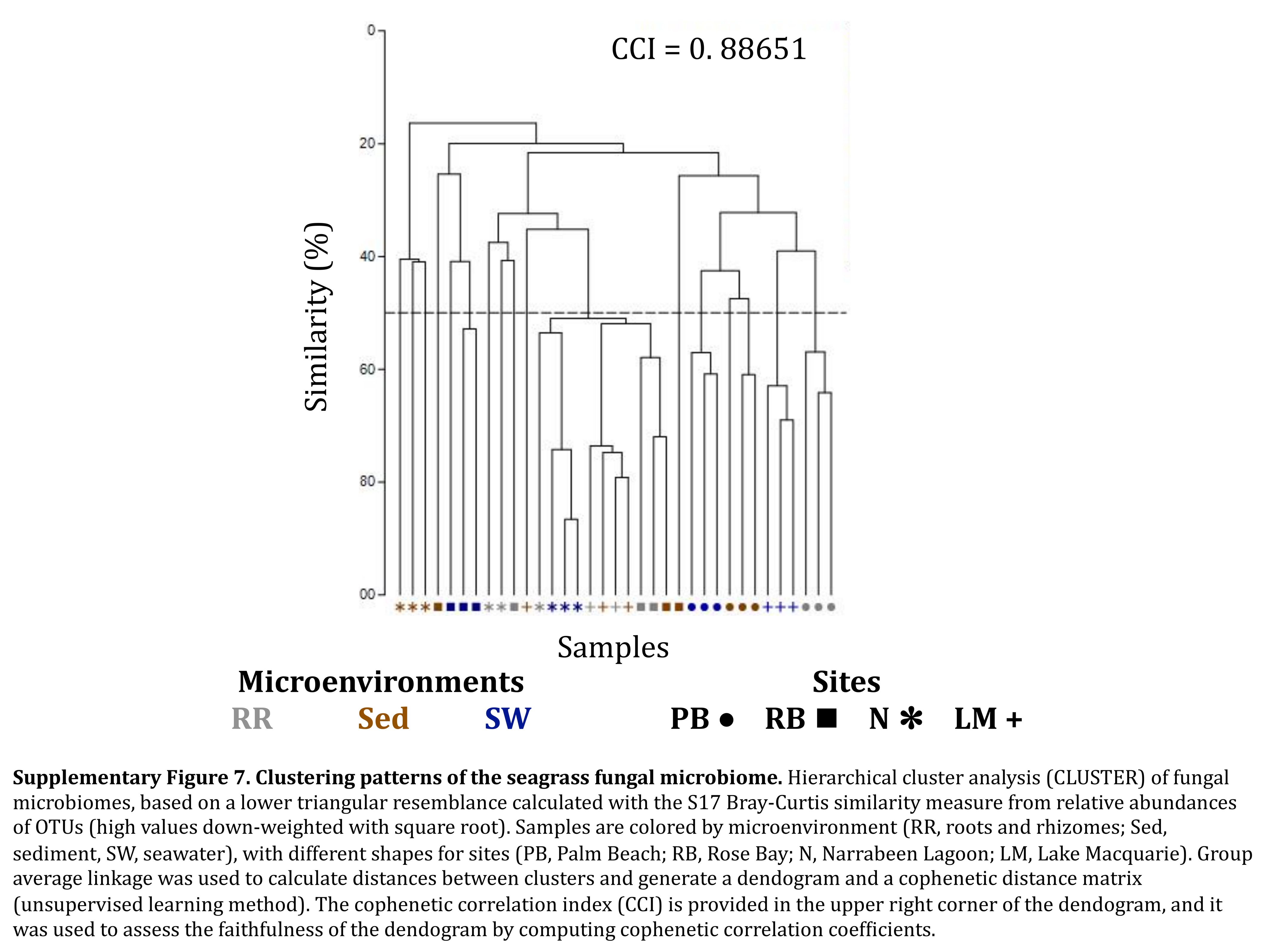

Supplement: Supplementary file 7 [file Image_7.JPEG]
